# Multiscale chromatin dynamics and high entropy in plant iPSC ancestors

**DOI:** 10.1242/jcs.261703

**Published:** 2024-06-24

**Authors:** Kinga Rutowicz, Joel Lüthi, Reinoud de Groot, René Holtackers, Yauhen Yakimovich, Diana M. Pazmiño, Olivier Gandrillon, Lucas Pelkmans, Célia Baroux

**Affiliations:** ^1^ Plant Developmental Genetics, Institute of Plant and Microbial Biology, University of Zurich, 8008 Zurich, Switzerland; ^2^ Department of Molecular Life Sciences, University of Zurich, 8050 Zurich, Switzerland; ^3^ Laboratory of Biology and Modeling of the Cell, University of Lyon, ENS de Lyon, 69342 Lyon, France

**Keywords:** *Arabidopsis*, iPSC, Protoplast, Chromatin dynamics, Texture features, Linker histone, High-throughput imaging, Supervised image analysis, Entropy

## Abstract

Plant protoplasts provide starting material for of inducing pluripotent cell masses that are competent for tissue regeneration *in vitro*, analogous to animal induced pluripotent stem cells (iPSCs). Dedifferentiation is associated with large-scale chromatin reorganisation and massive transcriptome reprogramming, characterised by stochastic gene expression. How this cellular variability reflects on chromatin organisation in individual cells and what factors influence chromatin transitions during culturing are largely unknown. Here, we used high-throughput imaging and a custom supervised image analysis protocol extracting over 100 chromatin features of cultured protoplasts. The analysis revealed rapid, multiscale dynamics of chromatin patterns with a trajectory that strongly depended on nutrient availability. Decreased abundance in H1 (linker histones) is hallmark of chromatin transitions. We measured a high heterogeneity of chromatin patterns indicating intrinsic entropy as a hallmark of the initial cultures. We further measured an entropy decline over time, and an antagonistic influence by external and intrinsic factors, such as phytohormones and epigenetic modifiers, respectively. Collectively, our study benchmarks an approach to understand the variability and evolution of chromatin patterns underlying plant cell reprogramming *in vitro*.

## Teaser

**Summary:** Plant protoplast cultures, similar to animal iPSC ancestor cell cultures, exhibit rapid, multiscale chromatin changes and a decreasing entropy during dedifferentiation.

## INTRODUCTION

Plant tissues have a remarkable plasticity. This phenomenon is illustrated by the capacity of plant parts, tissue fragments and isolated cells to regenerate whole plant individuals *in vitro.* This property has been exploited by horticulture and agriculture for centuries for the accelerated amplification of garden plants, crop and tree species, the propagation of disease-free plants, the production of plant biomass for industrial applications and the creation of starting material for genetic engineering approaches (Ibanez et al., 2020; Ikeuchi et al., 2019; Reed and Bargmann, 2021; Loyola-Vargas and Ochoa-Alejo, 2018). By contrast, organ regeneration, notably from single cells, is not a prevalent property in the animal kingdom, except in basal lineages such as Cnidarians (Holstein et al., 2003).

Cellular plasticity describes the ability of differentiated cells under certain conditions to reprogramme physiologically and molecularly towards a pluri-competent (or pluripotent) state (Reddy et al., 2021). There is a vivid interest in understanding the mechanisms controlling cellular plasticity. In animals, several pioneer transcription factors have been identified that can potentiate cell reprogramming following overexpression *in vitro* (Iwafuchi-Doi and Zaret, 2014). Similarly, several transcription factors have been identified in the model plant *Arabidopsis* that have tissue reprogramming properties [BABYBOOM, WUSCHEL, LEAFY COTYLEDON1 and WOUND INDUCIBLE 1 (reviewed in Ikeuchi et al., 2019; Iwase et al., 2017, 2011)]. Among them, so far only LEAFY has been formally demonstrated to share the molecular and cellular properties of a pioneer transcription factor (Jin et al., 2021). In addition, several studies concur with the idea that chromatin modifiers, controlling the epigenetic landscape and accessibility, are key to cellular plasticity, in both plants and animals (Birnbaum and Roudier, 2017; Reddy et al., 2021). For instance in *Arabidopsis*, mutants with reduced levels of DNA methylation or histone methylation (particularly H3K4me3, H3K27me3 and H3K9me2) have altered plasticity and have impaired or, by contrast, enhanced abilities for somatic embryogenesis, callus production, shoot or root regeneration or a combination thereof (He et al., 2012; Ishihara et al., 2019; Jing et al., 2020; Lee and Seo, 2018; Reddy et al., 2021; Shemer et al., 2015).

Conveniently in plants, excised root or shoot fragments, as well as isolated cells devoid of cell wall (protoplasts) provide starting material to generate pluripotent cells (Ikeuchi et al., 2019). When cultivated on medium supplemented with phytohormones, protoplasts dedifferentiate before re-entering the cell cycle. Proliferation then enables the formation of cell masses, microcalli, within which some cells will express pluripotency markers. These plant induced pluripotent stem cells (iPSCs) will differentiate into shoot and root tissues competent to form a fully fertile plant (reviewed in Muller-Xing and Xing, 2022; see also Fig. 8 in the Discussion). Protoplast cultures, which are considered to share ‘stem cell-like’ properties (Grafi et al., 2011; Sang et al., 2018), can thus be seen as the ancestors of plant iPSCs, similar to the ancestor cultures of animal iPSCs, which consist of cells released from animal tissues and destinated for *in vitro* reprogramming. Also, these plant and animal ancestor cultures both have a low efficiency (<0.5%) of iPSC production (Xu et al., 2021; Ghaedi and Niklason, 2019).

The release of protoplasts from their native tissue rapidly leads to transcriptome reprogramming, with a large fraction of affected genes corresponding to stress responses, energy metabolism and photosynthesis (Chupeau et al., 2013). However, this response is not uniform, even in cultures composed of 95% mesophyll cells, and the cultures are characterised by a high level of heterogeneity in the transcript composition among cells (Xu et al., 2021). Transcriptome reprogramming is accompanied by profound changes in chromatin accessibility (Xu et al., 2021; Zhao et al., 2001) and in histone acetylation, which is thought to establish a transcriptionally permissive landscape (Williams et al., 2003). Chromatin changes are also visible at the cytological level. Pioneer studies in tobacco and *Arabidopsis* (Tessadori et al., 2007; Williams et al., 2003; Zhao et al., 2001) have shown that heterochromatin decondenses during, or shortly after, protoplast isolation (Zhao et al., 2001), leading to the spatial dispersion of centromeric and pericentromeric repeats together with their associated DNA and histone methylation marks (Tessadori et al., 2007) and the decondensation of rDNA arrays (Ondrej et al., 2010). Despite these large-scale alterations, transposable elements and genomic repeats remain transcriptionally silent, suggesting uncoupling of heterochromatin condensation and silencing (Tessadori et al., 2007). Within the first 3–5 days of culture, heterochromatin gradually recondenses and the transcriptome changes again, showing more attributes of the cell cycle and regeneration process (Chupeau et al., 2013; Xu et al., 2021).

We aimed here to obtain a comprehensive, quantitative overview of the cytological patterns of chromatin (re)organisation during early culturing of protoplasts, corresponding to the dedifferentiation phase (Grafi et al., 2011). In order to foster conceptual comparisons between the cellular reprogramming phases leading to plant and animal iPSC generation, we will use the term plant iPSC ancestors to refer to the protoplast cultures. Specifically, we deployed high-throughput imaging and customised a supervised-learning image processing to analyse the chromatin patterns in leaf-derived iPSC ancestors within the first 5–7 days of culture. Multivariate analysis of the different chromatin features revealed rapid chromatin changes at different scales. The chromatin of plant iPSC ancestors also rapidly changed in composition, with a notable decrease in linker histone complements. We found that the trajectory of chromatin changes largely depended on nutrient availability and less on phytohormones. In addition, the cultures were characterised by a high heterogeneity as assessed by entropy analyses. However, the entropy of chromatin features decreases progressively in the following 5-to-7 days, a process dampened by the absence of phytohormones but enhanced by an inhibitor of histone deacetylation.

## RESULTS

### H1 as a marker of chromatin reorganisation at early stages of plant iPSC ancestor culture

**Fig. 1.**
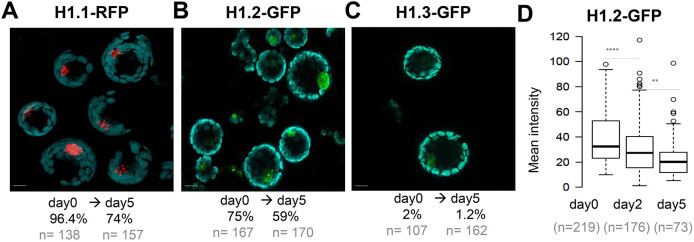
**Plant iPSC ancestor cultures are marked by a progressive decrease in H1.1 and H1.2 linker histone variants.** Protoplasts were prepared from *Arabidopsis* leaves expressing a fluorescently tagged variant of H1.1 (A), H1.2 (B) or H1.3 (C) and their level was assessed during 5 days of culture. (A–C) Representative images and percentage (%) of cells with detectable fluorescence signal (*n*=number of cells scored). See also source data in Table S1 and additional related measurements in Fig. S1. Cyan, chloroplasts (autofluorescence); red, RFP; green, GFP. (D) Measurements of H1.2–GFP signal intensity in a replicate culture sampled at day 0, 2 and 5. ****P<0.0001; **P<0.01 (Mann–Whitney *U*-test). Scale bars: 10 µm.

To select a live reporter monitoring chromatin changes, we analysed various fluorescently tagged histone variants. First, we considered the three *Arabidopsis* linker histone variants for which translational fusions are available (Rutowicz et al., 2019; 2015). In a first approach, we scored the number of fluorescence-positive cells at day 0 (just after isolation), and at day 2 and at day 5. At day 0, H1.1–RFP and H1.2–GFP were detected in most cells (96%, *n*=138 and 75%, *n*=168, respectively). However, at day 5 the fraction of detectable positive cells had decreased by ∼25% and 22%, respectively (Fig. 1A,B; Fig. S1A). By contrast, the stress inducible, H1.3–GFP variant reporter was detected only in 2% cells at day 0, likely corresponding to guard cells in which it is constantly expressed (Rutowicz et al., 2015). This fraction did not increase upon culturing (Fig. 1C; Fig. S1A). Thus, this marker was not further considered. In a second approach, we focused on H1.2, which is the most abundant variant in leaf cells (Kotlinski et al., 2016) and quantified the mean signal intensity per nucleus. We measured a 30% decrease of H1.2–GFP abundance in the fraction of expressing nuclei at day 5 (Fig. 1D).

In contrast, two core nucleosome histone reporters (H2B–RFP and H3.3–GFP, respectively) were equally detected throughout the culturing time (Fig. S1B). A GFP-tagged H2A.Z reporter also included in the analysis was not further considered as it captured only ∼40% cells (Fig. S1B).

Finally, to quantify the mitotic competence of the cultured cells in our conditions, we monitored a S-to-early G2 phase marker (Desvoyes et al., 2020). At day 0 only 5% cells (*n*=275) showed detectable signal, with this increasing to 8% (*n*=250) at day 5 and 11% (*n*=230) at day 6 (Fig. S1C). Thus, plant iPSC ancestor cultures are mitotically relatively quiescent in the first 6 days, which corresponds to dedifferentiation, the first phase of plant cells reprogramming (Grafi et al., 2011). Furthermore, the scarcity of S-phase occurrence cannot explain the major decrease in H1 abundance that had already occurred by day 3 (Fig S1A). This suggests that there is an active mechanism degrading H1 and likely contributing to the heterochromatin decondensation described previously in leaf protoplasts (Tessadori et al., 2007).

### A semi-automated pipeline for high-throughput analysis of chromatin reporters

**Fig. 2.**
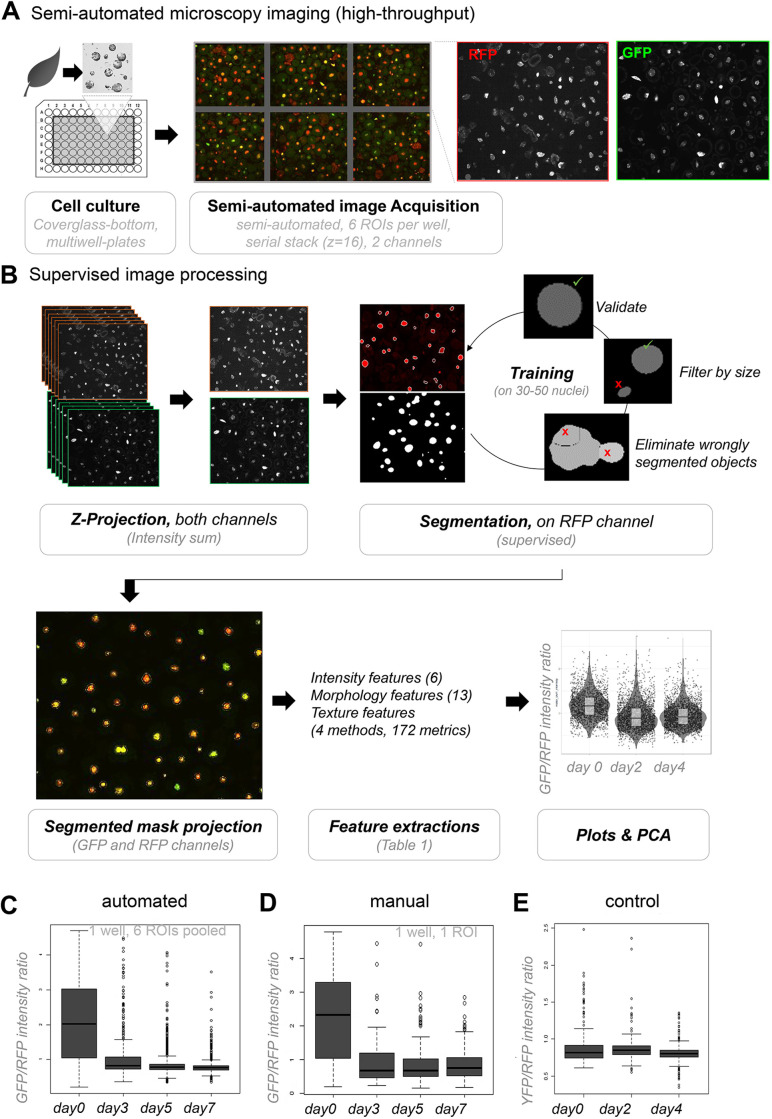
**A semi-automated pipeline for high-throughput analysis of chromatin reporters in plant iPSC ancestor cultures.** (A) Semi-automated microscopy imaging is carried out in cover-glass bottom multi-well plates containing cultures of protoplasts, plant iPSC ancestors, using a Cell Voyager platform. Six Regions of Interest (ROI) are randomly selected per well and imaged for each channel. The procedure is repeated for each time point, the culture being returned to the growth incubator in between two measurements. (B) Supervised image processing followed a workflow as depicted using TissueMAPS and enabled the segmentation of several hundred nuclei with high accuracy. The image analysis package returns several descriptors (features) of the segmented objects describing the morphology, the intensity distribution and texture (see details in the text). These metrics can be plotted or further analysed. (C,D) Comparison of the H1.2-GFP/H2B-H2B ratios obtained following automated (C) or manual (D) image analysis, showing a reproducible reduction of H1.2-GFP relative to H2B-RFP during culturing time (experiment HTI001; see Table S4). (E) Control experiment showing stable signals of a nuclear localised, free YFP marker, relative to H2B–RFP levels (experiment HTI002; see Table S4).

We aimed at high-throughput imaging of plant iPSC ancestors in culture, similar to what had been undertaken previously for analysing cellular morphology (Dawson et al., 2022), but focusing here on chromatin markers. For this, we generated a dual reporter *Arabidopsis* line expressing both the H1.2–GFP and H2B–RFP markers (selected following the strategy explained above), and used it to establish and benchmark the growth conditions, imaging setup and an image analysis pipeline. Details are provided in the Materials and Methods but, in brief, leaf protoplasts were cultured under sterile conditions in 96-well plates with a coverglass bottom for semi-automated imaging using Cell Voyager (Fig. 2A). The imaging set-up allows the capturing of up to 60 wells (i.e. 60 cultures) per multi-well plate, each being covered by six regions of interest (ROIs). Imaging of a full 60×6 ROIs takes 90 min. After each time point, the plate was returned to the temperature and light-controlled plant growth incubator until the next measurement. Image analysis, illustrated in Fig. 2B, consisted of a supervised, batch processing approach comprising the following steps performed in TissueMAPS (http://tissuemaps.org): (1) maximum intensity projection of the image series; (2) denoising; (3) watershed-based segmentation of H2B-RFP-stained nuclei and image masking using the segmented objects; (4) training, classification and filtering of segmented objects to improve nuclei segmentation; and (5) quantitative measurements using validated nuclei objects.

To benchmark the above approach, we cultured protoplasts expressing the dual markers in replicate wells and applied our imaging and image processing procedure between day 0 and day 7 (representative images are shown in Fig. S2A,B). The setup of replicate wells allowed the capture of a total of 500–1000 nuclei per time point, per initial culture. Using fluorescein diacetate (FDA) staining (Saruyama et al., 2013), we confirmed a high density of viable cells at each time points (Fig. S2C). However, we observed a decrease in the number of nuclei (cells) identified following the segmentation, particularly at day 7 (Fig. S2D), indicating loss of viability, consistently with previous reports (Chupeau et al., 2013; Xu et al., 2021). The analysis of GFP to RFP signal intensity ratios per nuclei confirmed a dramatic reduction of H1.2 abundance relative to H2B as soon as day 3 (Fig. 2C). This automated measurement compared very well to a set of manually segmented images used for the same intensity ratio measurement (Fig. 2D). Then, to assess the reproducibility of the culturing–imaging–image analysis workflow, we set up eight replicate measurements in the same multiwell plate with cultures from two independent reporter lines expressing H1.2–GFP and H2B–RFP. The signal intensity ratio distributions were highly consistent between wells at each day (Fig. S2E). Finally, as a control, we analysed cultures co-expressing a nuclear localised (NLS–YFP) and the same H2B–RFP internal control, using the same setup as before. The analysis showed a stable ratio during culturing (Fig. 2E; Fig. S2F).

In conclusion, we established a robust automated high-throughput imaging-image analysis workflow enabling the capture of several hundred nuclei per time point and which is suitable for quantitative measurements of chromatin patterns during culturing of plant iPSC ancestors. In addition, the quantifications confirmed that a decrease in H1.2 abundance is a hallmark of chromatin changes within the first 2 days of culturing.

### Nuclei morphology and chromatin patterns change rapidly

**Fig. 3.**
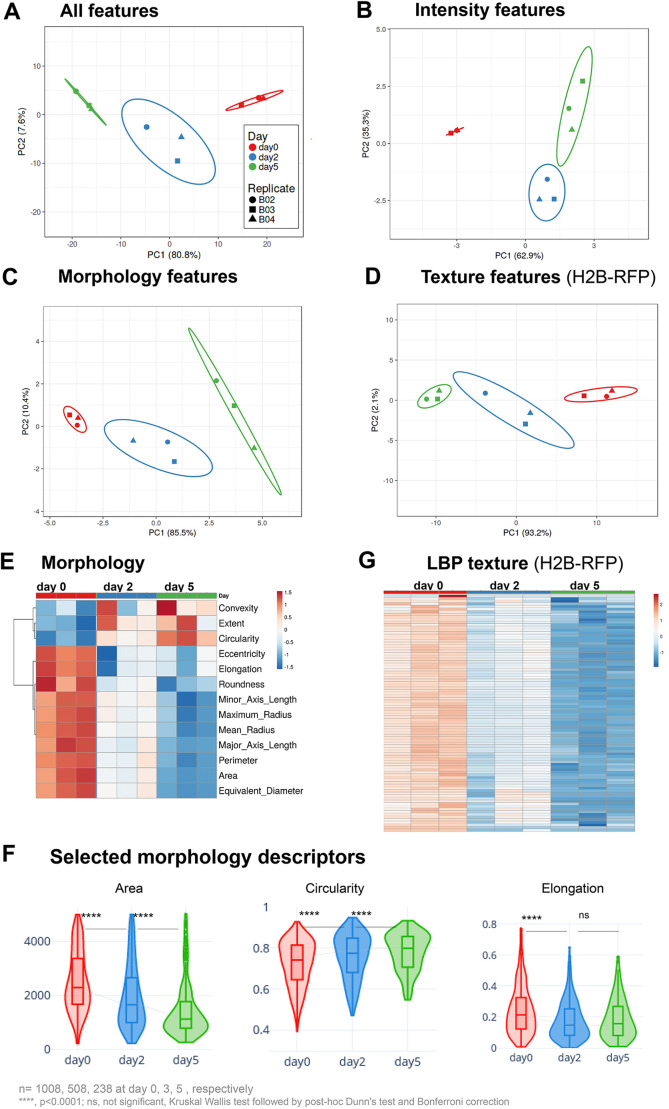
**Marked changes in nuclear morphology and chromatin organisation during the dedifferentiation phase.** (A–D) PCA of chromatin features measured in *Arabidopsis* leaf protoplasts expressing H1.2–GFP and H2B–GFP, imaged ∼4 h after release (day 0), at day 2 and day 5 (experiment HTI004; see Table S4). (A) PCA computed on all features (Table S2), (B) PCA on H1.2–GFP and H2B–GFP intensity features, (C) PCA on morphology features (i.e. size and shape descriptors of the segmented nuclei) and (D) PCA on LBP texture features for H2B–RFP. See also Fig. S3A for replicate PCAs and Fig. S3B for the PCA loading scores per descriptors. The *x*- and *y*-axis show principal component 1 and principal component 2 that explain the given percentage of the total variance, respectively. Ellipses: 95% confidence interval. Each point represents a culture replicate (well). (E) Relative changes for each morphology descriptors during culturing. Heatmaps represent the median value for each descriptor, with unit variance scaling applied to rows. (F) Plots of selected morphology descriptors. Data is shown as a violin plot, with a box plot inside, where the box represents the 25–75th percentiles, and the median is indicated as a line. The whiskers show the minimum and maximum values, respectively. (G) Relative changes for LBP texture descriptors for the H2B–RFP signal distribution. Heatmaps represent the median value for each descriptor, with unit variance scaling applied to rows. The list of LBP descriptors (rows) is provided in Table S2. Rows are clustered using correlation distance and average linkage. Number of nuclei, *n*=1008, 508, 238 at day 0, 3, 5, respectively. ****P<0.0001; ns, not significant (Kruskal–Wallis test followed by post-hoc Dunn's test and Bonferroni correction).

Next, we exploited the numerous image features exported by the pipeline to analyse the cytological organisation of chromatin in plant iPSC ancestors, particularly during the first days of culturing corresponding to the dedifferentiation phase, before cells start dividing (Grafi et al., 2011). Three groups of metrics were produced from the analysis – signal intensity features, morphology features and texture features. This corresponds to a total of 193 metrics per channel and 370 for both channels (morphology features were derived from segmentation on the H2B–RFP signal only, Table S1). A principal component analysis (PCA; Metsalu and Vilo, 2015) that included all the features indicated clear changes within the first 2 days of culturing (Fig. 3A, a replicate is shown in Fig. S3A).

To then estimate the contribution of each feature group, we carried out separate PCA. Clearly, each of the intensity, morphology and texture features contributed to the explanation of the chromatin organisation changes during the dedifferentiation phase (Fig. 3B–D; Fig. S3A).

The observation that intensity features distinguish cells at day 0, day 2 and day 5 indicates that the relationships between chromatin markers change rapidly in the early culturing phase. Consistent with our previous observation that cells barely divide within the first 5 days of culturing, we found that the H2B–RFP intensity distribution did not significantly change during this period of time, but that it had increased by day 7, probably indicating that cells had entered S phase (Fig. S3B). It is likely that the global decrease in linker histone abundance (H1.2–GFP), relative to total chromatin (H2B–RFP), as documented above, has a major contribution to the separation of chromatin features on this PCA. However, the standard deviation, and minimum and maximum intensity values also contribute the principal components (Fig. S3C) suggesting that, beyond the absolute levels of chromatin markers, their spatial distribution, reflecting the occurrence of chromatin regions with varying density and compaction, also change rapidly.

Morphology features are computed on the segmented H2B–RFP signal, and thus provide a proxy for nuclei size and shape (Fig. S3D). A PCA considering all morphology features indicated that nuclei undergo continuous size and shape changes between day 0 and day 5 (Fig. 3C,E). Interestingly, although an increase in nuclear size would be expected from the decreased abundance in H1 variants (Rutowicz et al., 2019; She et al., 2013), we did not detect a positive correlation, apart from a moderate one at day 5 (Pearson correlation *r*<0.3, Fig. S3E). Instead, the nuclear size distribution shifted towards smaller sizes at day 2 and day 5 (Fig. S3F). The median and the distribution of shape descriptors at day 5 also differed from that at day 0 (Fig. 3E), with, for instance, slightly rounder and less elongated nuclei at day 5 than day 0 (Fig. 3F).

Next, we interrogated the group of texture features, which showed a clear evolution during culturing (Fig. 3D). Texture metrics describe the spatial distribution of signal intensities as a function of scale (Depeursinge et al., 2017; Di Cataldo and Ficarra, 2017) and can be used to analyse patterns in chromatin organisation (Kerr et al., 2010; Lee et al., 2021; Rana et al., 2021). TissueMAPS returns metrics corresponding to four types of texture analysis: Gabor wavelet filter, local binary pattern (LBP), threshold applied statistics (TAS), and Hu invariant moment (Hu) (reviewed in Di Cataldo and Ficarra, 2017; Hamilton et al., 2007; see the list in Table S2). The LBP analysis, based on neighbouring pixel intensity scanning in incrementally growing circles, is particularly interesting as a proxy of chromatin organisation patterns at different length scales (Fig. S3G) (Di Cataldo and Ficarra, 2017; Rana et al., 2021). We detected a rapid, global decrease in LBP values along the different radii for the H2B–RFP signal (Fig. 3G) suggesting a decreasing heterogeneity in chromatin distribution at day 2. The texture metrics of the H1.2–GFP signal also showed rapid changes in iPSC ancestor chromatin (Fig. S3G). These dynamics in H2B–RFP and H1.2–GFP textures, reflecting the distribution pattern of these histone variants, were confirmed with the TAS and Gabor filter methods (Fig. S3H,I). Furthermore, we detected modest, but consistent positive correlations between the LBP metrics and the H1.2–GFP-to-H2B–RFP ratio, indicating that the changes in chromatin distribution patterns are likely to be linked with the relative abundance of linker and nucleosome histones (Fig. S3J).

Collectively, the analysis of nuclei morphology and of H2B–RFP signal distribution indicate clear changes in nuclear size, shape and in chromatin organisation in the dedifferentiation phase of plant iPSC ancestors (Grafi et al., 2011). These changes occur largely within the first 2 days and are concurrent, but not correlated, with a decrease in the relative abundance of linker histone H1.2. The distinct texture of H2B–RFP distribution at different length scales at day 5 compared to day 0 suggests a reorganisation of chromatin domains at the (sub)micrometre scale.

### Plant iPSC ancestor cultures show a marked heterogeneity in their chromatin features, which reduces over time

**Fig. 4.**
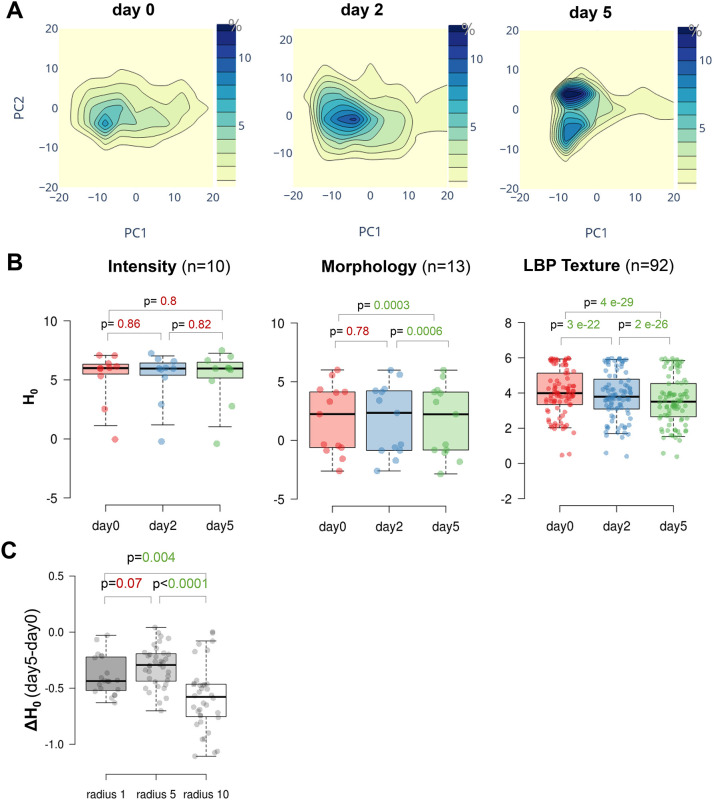
**Plant iPSC ancestor cultures are characterised by a high entropy of chromatin features, reducing over time.** (A) Density distribution of chromatin features summarised by principal components PC1 and PC2 as computed in Fig. 3A. Density contours are coloured according to the frequency (percentage) of nuclei falling in the corresponding PC space. (B) Entropy (H_0_) of chromatin features per family (*n*, number of descriptors per family). (C) Differential entropy (ΔH_0_) between day 5 and day 0 for LBP texture features of H2B-RFP and at different length scale (radius). *P* values, paired Wilcoxon rank test. For box plots, the box represents the 25–75th percentiles, and the median is indicated. The whiskers show the minimum and maximum values. Data is from experiment HTI004; see Table S4.

The above analysis clearly showed a spread distribution of intensity, size and shape descriptors, indicative of a vast heterogeneity of nuclei type, despite the fact that leaf-derived protoplasts consist of ∼85–90% mesophyll cells (Xu et al., 2021). To assess this heterogeneity, we first generated density distribution maps of chromatin features summarised by the principal components (PCs) computed previously. The maps confirmed a broad dispersion of the data in the PC landscape, indicating a very heterogenous population in terms of nuclei type and chromatin patterns (Fig. 4A). The distribution however changed over time with an apparent enrichment of nuclei with similar PC values at day 5 (Fig. 4A; Fig. S4).

To quantify this heterogeneity and possible changes during the culturing time, we computed the entropy of the data. Entropy is a useful measure of variability in biological data, capturing both the variance and the shape of the data distribution (Gandrillon et al., 2021). The analysis indeed revealed a marked positive entropy (H_0_) for a large fraction of chromatin features (Fig. S4B), changing significantly over time.

We then analysed the family of features separately and found that heterogeneity as assessed by a positive entropy was contributed to by all features (Fig. 4B). Strikingly, the entropy decreased over time, particularly that of texture features and moderately for morphology features, whereas it remained largely positive for intensity features. Interestingly, the heterogeneity of chromatin distribution, measured by LBP texture features on H2B–RFP, was higher at a small length scale (LBP radius 1, Fig. S4C) but entropy reduction over time (ΔH_0_) was more significant for the highest length scale (radius 10, Fig. 4C).

These findings were confirmed in a replicate experiment and where an additional imaging time point, at day 7 showed that entropy continue to decrease, but more slowly, after day 5 (Fig. S4D).

In conclusion, entropy analysis indicates a profound heterogeneity of nuclei morphology and of chromatin organisation among iPSC ancestors. Strikingly, heterogeneity reduces progressively, mostly within the first 5 days, and more particularly for chromatin distribution patterns (texture). This suggests a tendency towards homogenisation of chromatin types, although entropy remains high even after 7 days.

### Chromatin heterogeneity is influenced by phytohormones

**Fig. 5.**
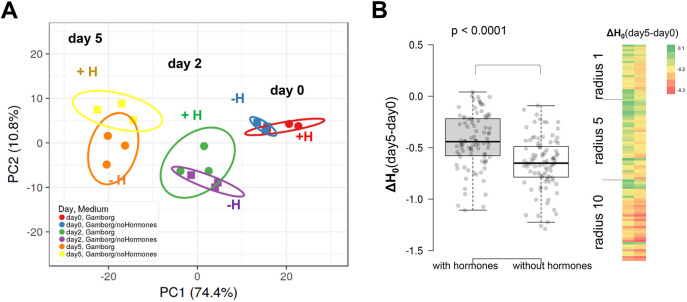
**Phytohormones do not influence the trajectory of chromatin changes induced by culturing but dampen entropy reduction.** (A) PCA of chromatin features of plant iPSC ancestor cultured in the Gamborg medium, including phytohormones (‘Gamborg’ or ‘+H’ as quick annotation on the graph), or in the same basis but without phytohormones (‘Gamborg/no Hormones’ or ‘-H’ as quick annotation on the graph). The PCA was computed with all features (see Materials and Methods, data is from experiment HTI004; see Table S4). (B) Differential entropy (ΔH_0_) between day 5 and day 0 for the chromatin texture features (LBP texture metrics, H2B-RFP) of cells cultured in Gamborg with or without hormones as indicated. *P* value: Wilcoxon signed-rank test. The box represents the 25–75th percentiles, and the median is indicated. The whiskers show the minimum and maximum values.

Protoplast cells cultured in the absence of phytohormones do not grow nor do they divide and undergo progressive cell death (Zhao et al., 2001). We thus asked whether the chromatin changes detected within the first days of culturing are part of the cellular responses to phytohormones. For this, we partitioned the initial pool of freshly released cells in two media – either in the regular Gamborg B5's medium (Gamborg et al., 1968), which is rich in macro- and micro-elements, vitamins, and is supplemented with glucose (2%) and phytohormones (auxins and cytokinin), or in the same medium but without phytohormones. First, we asked whether the absence of phytohormones would affect the reduction in the relative abundance of linker histones (H1.2) that was observed above. Quantifications showed that this is not the case and that H1.2 reduction still took place in the absence of phytohormones, although perhaps along a milder gradient (Fig. S5A). This suggests that H1.2 reduction is not a response to phytohormones in the medium but most likely a response to the cellular isolation, away from the source tissue, and culturing. Next, we interrogated the entire family of chromatin features with or without hormones, using PCA. The analysis showed that cells cultured without hormones underwent similar changes in chromatin organisation to that seen in the presence of hormones (Fig. 5A; Fig. S5B). However, and unexpectedly, we detected that chromatin organisation heterogeneity (measured by the entropy on the LBP texture of H2B–RFP distribution) decreased significantly more in the absence of phytohormones (Fig. 5B), whereas the heterogeneity of intensity and morphology features were not affected or were only moderately affected (Fig. S5C). We made the same observation when culturing the cells in a nutrient-poor medium (W5) without phytohormones (Fig. S5D). This suggests that phytohormones contribute to maintaining a certain level of heterogeneity during dedifferentiation in the plant iPSC ancestor cultures. Whether the effect is direct, with chromatin reorganisation responding to phytohormones, or indirect, owing to higher cell viability in the presence of phytohormones (Fig. S1D), remains to be determined. However, the first scenario is supported by the numerous evidence of crosstalk between phytohormones and chromatin modifiers particularly affecting plant cell identity and plasticity (Maury et al., 2019).

### Nutrient availability influence chromatin changes during culturing

**Fig. 6.**
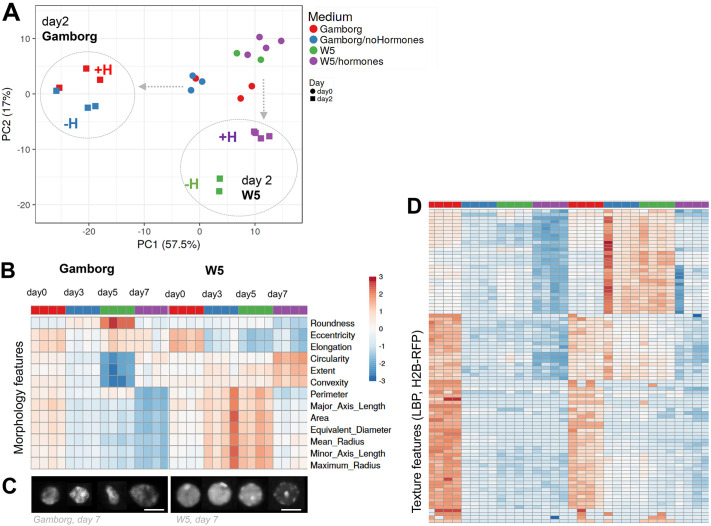
**Nutrient availability strongly influences the trajectory of chromatin changes in plant iPSC ancestor cultures.** (A) PCA of chromatin features in cells cultured either in a nutrient rich (Gamborg) or in a nutrient poor (W5) medium, each with (+H) or without (−H) hormones. The cultures stem from the same, original pool of protoplasts partitioned in the different media and were imaged at day 0 and day 2 in two or three replicate wells (number of datapoint with the same colour). Data is from experiments HTI004 and HTI005; see Table S4). (B) Morphological features of nuclei from cultures either in Gamborg or W5 medium, at day 0, 3,5 and 7 (data is from experiment HTI001; see Table S4), each square unit is a replicate well. Median values are normalised (centred by rows) and the colour scale shows the fold change. (C) Representative nuclei of the same experiment, day 7. Scale bars: 5 μm. (D) Chromatin texture features (LBP method, H2B–RFP signal) of the same culture as in B (labels and key are the same) showing comparatively stronger changes between day 0 and day 2 in Gamborg compared to in W5. The list of LBP descriptors (rows) is provided in Table S2. Rows are clustered using correlation distance and average linkage.

To answer the question of whether chromatin changes respond to the physiological quality of the culturing medium we partitioned a pool of freshly released leaf protoplasts into W5 (nutrient poor; Yoo et al., 2007) or Gamborg B5 (nutrient rich; Gamborg et al., 1968), both prepared with or without phytohormones (Table S3). We analysed the chromatin features as before focusing on the first 2 days. Clearly, the culturing medium strongly influenced the chromatin changes with distinct trajectories principally influenced by the nutrient basis more than by the phytohormones (Fig. 6A; Fig. S6A).

Next, to verify whether the nutrient composition influences chromatin changes further in time we imaged new cultures for 7 days. The analysis confirmed that major changes occur essentially between day 0 and day 3, yet according to different trajectories depending on the medium, and with chromatin features stabilising rapidly after day 3 (Fig. S6B). The typical process of H1.2 reduction also occurred in the nutrient-poor medium (W5) although at a slightly lower rate (Fig. S6C). The medium affected nuclei morphology (Fig. 6B; Fig. S6D) with notably rounder and larger nuclei in the nutrient-poor (W5) medium (Fig. 6C; Fig. S6D,E). The nutrient-poor medium also dampened changes in chromatin texture across all length scales, possibly indicating a slower transition in chromatin reorganisation (Fig. 6D).

Finally, we asked whether the heterogeneity of chromatin features was affected by the culturing medium. The differential entropy (ΔH_0_) between day 0 and day 7 per family of features did not reveal an overall significant effect of the medium (Fig. S6F), although, some morphology features showed an increase (e.g. area) or a decrease (e.g. roundness) in entropy in the nutrient-poor medium compared to the Gamborg's medium (Fig. S6G).

In conclusion, nutrients strongly influenced chromatin dynamics during the dedifferentiation phase of plant iPSC ancestor culture, with lower nutrient availability inducing rounder, bigger nuclei with a less differentiated chromatin texture.

### Trichostatin A, an inhibitor of histone deacetylation increases heterogeneity of chromatin patterns

**Fig. 7.**
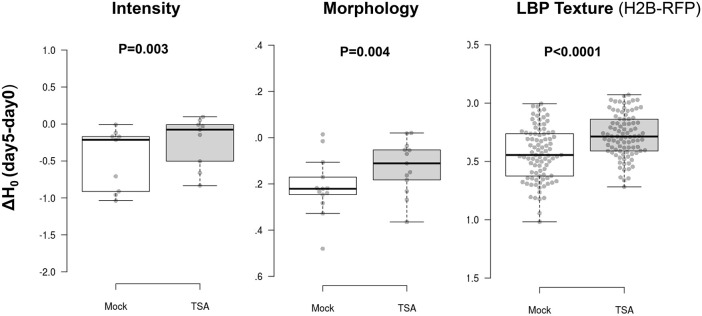
**TSA prevents entropy reduction of chromatin features.** Differential entropy (ΔH_0_) between day 5 and day 0 for families of chromatin features as indicated, comparing cells cultured in the Mock medium (Gamborg complemented with 2% DMSO) or in the same medium supplemented with 200 nM TSA in DMSO. Dataset data is from experiment HTI004; see Table S4). *P* values: Wilcoxon signed-rank test. The box represents the 25–75th percentiles, and the median is indicated. The whiskers show the minimum and maximum values.

Next, we tested the influence of trichostatin A (TSA), a compound known to inhibit histone deacetylases and increase histone acetylation (Yoshida et al., 1995). TSA treatment was shown to enhance the regenerative competence of lettuce and *Nicotiana* protoplast cultures, notably inducing a higher rate of division starting from day 5 (Choi et al., 2023). We rationalised that this drug could possibly accelerate chromatin decondensation or alter the chromatin feature dynamics that were detected with our approach. A global analysis using PCA did not reveal a major influence of TSA (Fig. S7A). In addition, TSA did not prevent nor accelerate H1.2 reduction, a typical event of the first culturing day, but did cause a small fraction of cells to maintain a high H1.2-to-H2B ratio at day 5 (Fig. S7B). The treatment also moderately influenced nuclei size but not shape (Fig. S7C). It is possible that chromatin acetylation, as described previously in tobacco protoplasts (Williams et al., 2003), is so rapid that TSA might act redundantly with the endogenously driven process of chromatin decondensation. However, when we measured the entropy of chromatin features, and more specifically the differential entropy between day 5 and day 0, we observed a strong effect of the treatment. Indeed, TSA abolished or strongly diminished the differential entropy for most features (Fig. 7A), indicating that TSA-treated cultures maintained a high level of heterogeneity at day 5. Hence, histone deacetylation might contribute to channel chromatin reorganisation during the first days of culturing.

## DISCUSSION

**Fig. 8.**
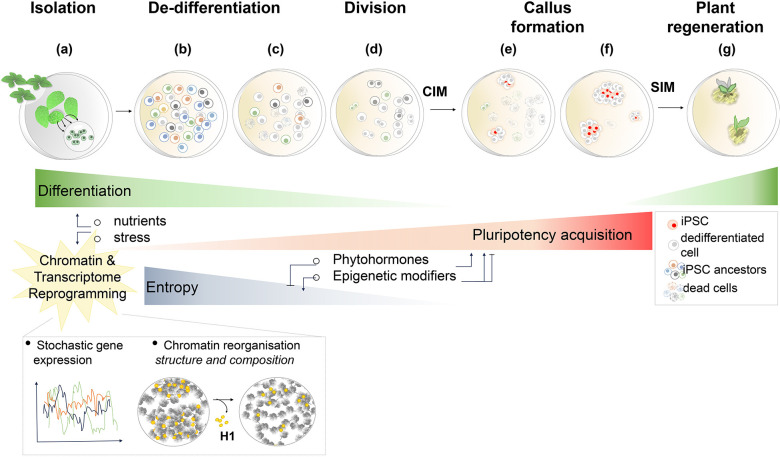
**Plant iPSC ancestor cultures are highly heterogenous, with entropy decreasing during early reprogramming.** Working model proposing a role for heterogeneity, at the gene expression and chromatin organisation level, in pluripotency acquisition during *in vitro* plant cell reprogramming. This model is proposed based on this work and that of others cited in the main text. Hypothetical extrapolations are made to offer a conceptual framework for future investigations. (a) Plant cells devoid of cell walls, called protoplasts, are isolated for instance from shoot tissues (or from other plant organs, not shown here). Protoplasts undergo a phase of dedifferentiation in culture associated with massive transcriptome reprogramming and chromatin reorganisation occurring at multiple scales. Notably, depletion of linker histones (H1) likely impacts structural and epigenetic rearrangement of chromatin domains; H1 depletion affects a large fraction of cells, but not all. (b) The initial cell cultures are highly heterogenous (represented with cells of different colours), characterised by a high entropy in chromatin patterns (this work) and stochastic gene expression (Xu et al., 2021), likely induced by the culturing conditions where nutrients and induced stressed contribute. (c) In the first culturing days, during the dedifferentiation phase, cellular heterogeneity progressively decreases, and the trajectory depends on nutrient availability; while some cells undergo reprogramming other perish (cells in dashed lines). Entropy (heterogeneity) is influenced by both extrinsic and intrinsic factors, antagonistically – phytohormones have a positive influence on cellular heterogeneity in the culture (the absence of phytohormones accelerate entropy decrease); by contrast, histone deacetylation enables the decrease in cellular heterogeneity, potentially contributing to the stabilization of chromatin structure and gene expression patterns]. Whether other epigenetic modifiers act as negative or positive regulators of heterogeneity remains to be investigated. (d) After 6–7 days, cells progressively re-enter the cell cycle and (e,f) form upon transfer on a callus induction medium (CIM) pluripotent cell masses. Callus cells expressing typical markers of shoot (or root) stem cells, correspond to induced pluripotent stem cells (iPSCs) by analogy to animal iPSCs. (g) Transfer on a shoot inducing medium (SIM) allows shoot regeneration initiated by the plant iPSC (similarly, roots can be produced from iPSC upon transfer on a root inducing medium, not shown here). In a broad sense, protoplasts can be considered the ancestors of plant iPSCs, following reprogramming induced by the culturing conditions, similar to what occurs in animal somatic cell cultures that are the ancestors of animal iPSCs following reprogramming induced by specific molecular factors. The process of induced cell pluripotency shares some common principles in both plant and animal model systems – pluripotency acquisition is largely inefficient (<0.5%) and starting cultures are characterised by a high cellular heterogeneity decreasing over time. This collectively suggests that pluripotency might arise from a population-based, statistical property rather than a single-cell competence (MacArthur and Lemischka, 2013).

Protoplast cultures offer numerous applications in plant sciences, covering both fundamental and applied research, from the elucidation of molecular and biochemical processes in plant cells to the deployment of new molecular plant breeding approaches (Xu et al., 2022). They also provide an attractive model to study cellular reprogramming in plant model systems; following release, protoplasts undergo a phase of dedifferentiation (5–7 days) prior to re-entering a phase of cell division, which, under an appropriate culturing medium containing phytohormones, can lead to the formation of pluripotent cell masses competent for tissue and plant regeneration (Ikeuchi et al., 2019) (Fig. 8). Protoplasts, are therefore proposed to share a ‘stem-cell like state’ (Grafi et al., 2011) and can be considered as plant iPSC ancestors, a term conveniently offering a conceptual parallel with animal cells reprogrammed towards an iPSC fate. However, not all cells are competent for transdifferentiation and regeneration (Pasternak et al., 2020; Sugimoto et al., 2011). In fact, similar to the low efficiency of animal iPSC production (e.g. 0.01 to 0.1% for human iPSCs; Ghaedi and Niklason, 2019), the frequency of cells with regenerative potential was estimated at ∼0.5% in an *Arabidopsis* leaf-derived protoplast culture (Xu et al., 2022).

In the initial dedifferentiation phase, protoplasts – plant iPSC ancestors – undergo extensive transcriptome reprogramming and large-scale chromatin reorganisation (Chupeau et al., 2013; Moricova et al., 2013; Tessadori et al., 2007; Williams et al., 2003; Xu et al., 2021; Zhao et al., 2001). Surprisingly, even in a relatively homogenous culture composed of 85% leaf mesophyll-derived cells, gene expression changes appear largely stochastic (Xu et al., 2022), raising the question of the level of heterogeneity of cellular states in the plant iPSC ancestor cultures.

Here, we established and validated a semi-automated pipeline for high-throughput quantitative analysis of chromatin reporters at the single-cell level, allowing a detailed analysis of the level of heterogeneity of chromatin patterns in plant iPSC ancestor cultures and their dynamic changes during the dedifferentiation phase.

### Harnessing the informative potential of image features provide a new perspective on chromatin organisation in plant cells

Semi-automated high-throughput imaging, followed by supervised image segmentation, allowed us to record several hundreds of nuclei per day of observation, in 2–4 replicate cultures per experiment. Image analysis computed by TissueMAPS (http://tissuemaps.org) returned three family of features describing chromatin organisation: morphology descriptors of nucleus size and shape (the nucleus is the segmented object based on the H2B-RFP signal); intensity variables for each of the two, jointly expressed, chromatin markers consisting of fluorescently labelled histones H2B and H1.2, respectively; and texture descriptors for the distribution of chromatin markers.

Morphology and intensity features are commonly used to describe characteristics of segmented cells or nuclei because they are intuitive. Beyond the size, shape descriptors inform here on the roundness, elongation, convexity or solidity of the nuclei, which can be apprehended collectively in a multivariate analysis. Intensity features, in addition, inform on the absolute levels of each chromatin marker (intensity sum), their global compactness or density in the nucleus (intensity mean) and their intensity variability in the segmented object (standard deviation, minima and maxima). By contrast, texture features give abstract representations of signal distribution that cannot intuitively be assigned to a particular distribution pattern, that is, a chromatin phenotype in our case. However, those are useful to identify structured and repeated patterns (Depeursinge et al., 2017; Di Cataldo and Ficarra, 2017). Here, we focused on the LBP method (local binary pattern), which computes a local representation of signal distribution, based on pixel neighbourhood analysis, along circles of incrementally bigger radius, and scanning a growing number of positions along each circle (Ojala et al., 2001). The higher the LBP values at a given radius, the more heterogeneous the signal distribution, hence, this method informs on chromatin structures with contrasted density at different length scales. This approach has been reported to be powerful to classify the chromatin types from healthy versus carcinogenic cells presenting different aggregation phenotypes (Rana et al., 2021; Venkatachalapathy et al., 2021). Texture analysis indirectly informs on the homogeneity versus aggregation of chromatin at the mesoscale (determined by the LBP method scanning along window of different pixel size) and conceivably also captures the relative proportions of chromatin masses versus interchromatin compartment (Cremer et al., 2020). This approach thus nicely complements former cytological observations describing the disaggregation of the relatively large-scale heterochromatin domains at day 0 of protoplast cultures and their progressive reassembly (Ondřej et al., 2009; Tessadori et al., 2007; Williams et al., 2003; Zhao et al., 2001). We confirmed here that texture features allowed us to capture changes in chromatin organisation at different length scales (see below).

### The rapid, multiscale reorganisation of chromatin along a trajectory is mostly affected by nutrient availability rather than phytohormones

The release of protoplasts from plant tissues induces dramatic chromatin disorganisation, with the disassembly of heterochromatin domains being a landmark confirmed in different species (Ondřej et al., 2009; 2010; Tessadori et al., 2007; Williams et al., 2003; Xu et al., 2021; Zhao et al., 2001). Culturing induces a progressive reassembly of heterochromatin domains and re-entry in the cell cycle (starting at day 4 and beyond) in the presence of phytohormones (Tessadori et al., 2007; Williams et al., 2003; Xu et al., 2021; Zhao et al., 2001). Here, high-throughput imaging allowed us to capture additional characteristics of chromatin dynamics, in particular of heterogenous cultures and the role of culturing media. Multivariate analysis identified a clear shift in chromatin features between day 0 and days 2–3, both when considering all families of features (morphology, intensity and textures) or each separately. This indicates that chromatin reorganisation occurs rapidly, early during the dedifferentiation phase, and is detectable at multiple scales. Specifically, changes were most prominent for nuclear morphology and for the distribution of H1.2 and H2B as revealed by chromatin texture.

Strikingly, nutrient availability, more than the phytohormones, had an influence on the trajectory of chromatin changes. Nutrient mostly affected nuclear morphology and chromatin textures, with H1.2 reduction being largely unaffected. Low nutrient availability prevents protoplasts from re-entering the cell cycle and eventually leads to cell death upon prolonged culturing (Zhao et al., 2001). Here, we show that low nutrient availability has an immediate effect and led to larger, rounder nuclei with less structured chromatin (i.e. displaying low textures), right up until day 5–7. This is reminiscent of the situation in animal iPSC ancestors, where nutrients have a profound impact on chromatin dynamics during cellular reprogramming (Apostolou and Hochedlinger, 2013; Lu et al., 2021). Metabolic fluxes are thought to influence the availability of metabolites used in epigenomic modifications both in plant and animal cells (Apostolou and Hochedlinger, 2013; Lindermayr et al., 2020; Lu et al., 2021, 2023), up to the point where energy metabolism has been shown to influence cell fate decisions (Ly et al., 2020). Here, the nutrient-rich and nutrient-poor media used differed not only in the amount of micro- and macro-nutrients, but also in sugar availability. Glucose levels have been shown to control, via the TOR signalling pathway, the cytoplasmic-to-nuclear ratio of Polycomb-group repressive complex 2 (PRC2) components influencing H3K27me3 deposition in plants (Ye et al., 2022). H3K27me3 reprogramming is essential for the acquisition of pluripotency in tissue explants primed for callus development *in vitro* (He et al., 2012). Given the ground role of H3K27me3 in cell identity and pluripotency in multicellular organisms, it is tempting to speculate that plant iPSC ancestors might undergo H3K27me3 reprogramming within the first culturing days, corresponding to the dedifferentiation phase (Grafi et al., 2011), under the joint influence of H1 linker histones (Rutowicz et al., 2019; She et al., 2013; Teano et al., 2023) and sugar and/or nutrient availability (Lu et al., 2023; Ye et al., 2022). That other nutrients influence chromatin reorganisation is nevertheless likely given the dramatic impact of nutrient availability on the trajectories of chromatin features.

### A role of linker histone in plant cell dedifferentiation?

The pronounced decreased abundance of both linker histone variants H1.1 and H1.2 within the first culture days suggests that there are large-scale changes in chromatin composition, which might explain the enhanced chromatin accessibility previously measured (Xu et al., 2021; Zhao et al., 2001). Yet, interestingly, while reduced H1 levels correlate with chromatin decompaction and increased nuclear size in differentiated tissues (Rutowicz et al., 2019), this was not the case here in leaf-derived protoplasts. This suggests that there are mechanisms controlling nuclear size counteracting the possible effect of chromatin decondensation in protoplasts. In *Arabidopsis*, H1 abundance largely influences the levels and genomic distribution of DNA methylation, the levels of epigenetic marks such as H3K27me3, H3K4me3 and histone acetylation, heterochromatin domains and genome topology (Bourguet et al., 2021; Choi et al., 2020; He et al., 2024; Rutowicz et al., 2019; 2015; Teano et al., 2023; Wierzbicki and Jerzmanowski, 2005; Zemach et al., 2013). Whether the decrease of H1 levels observed here during dedifferentiation *in vitro* triggers vast epigenomic changes could therefore be expected, but remains to be explored.

A link between linker histones, chromatin reprogramming and pluripotency has been established in animal cells. On the one hand, gradual and vast changes are observed in the epigenetic landscape, chromatin accessibility and genome topology of animal cells undergoing iPSC reprogramming *in vitro* (Pelham-Webb et al., 2020). Besides, the abundance and type of linker, histone variants clearly contribute to several epigenomic and topological features of the genome (Fyodorov et al., 2018). On the other hand, depletion in somatic H1 variants is necessary for *in vivo* pluripotency acquisition in mouse primordial germ cells (Christophorou et al., 2014), and *in vitro* iPSC reprogramming is enhanced when an oocyte-specific H1 variant (H1foo) is expressed together with the traditional Oct4, Sox2 and Klf4 reprogramming factors (Kunitomi et al., 2016). Thus, it would be interesting to determine in the future whether depletion of canonical linker histone variants plays a role in the reprogramming competence of plant iPSC ancestor cultures and whether non-canonical variants, like H1foo in animals, exist that might facilitate this process. In addition, a comprehensive overview of epigenome reprogramming and topological reorganisation covering the entire process of plant cell dedifferentiation and pluripotency acquisition remains to be established, with single-cell resolution to account for the high cellular heterogeneity.

### Chromatin features of plant iPSC ancestors show a high entropy, which reduces over time and is antagonistically modulated by phytohormones and histone deacetylation

Transcriptome analyses of protoplast cultures from different plant species have shown that these have extensive reprogramming compared to their source tissue (Cápal and Ondřej, 2014; Chupeau et al., 2013; Xu et al., 2021). Yet recently, single-cell-based reconstructions have demonstrated a high heterogeneity of transcriptome patterns even in a relatively homogenous protoplast culture composed of 85% mesophyll cells (Xu et al., 2021). Cell-to-cell variability, particularly in animal systems, has been recognised as an important factor controlling the inherent properties of a cellular system prompting investigations to understand its origin and regulation (Eldar and Elowitz, 2010; Eling et al., 2019; Guillemin and Stumpf, 2021; Huang, 2009; Mitchell and Hoffmann, 2018; Mojtahedi et al., 2016; Pelkmans, 2012; Richard and Yvert, 2014; Safdari et al., 2020). One approach to measure the heterogeneity, or information content, of biological systems is based on the calculation of the Shannon entropy, as borrowed from statistical mechanics (Gandrillon et al., 2021; MacArthur and Lemischka, 2013).This has been successfully applied to measure variability versus robustness of gene expression during cell differentiation (Dussiau et al., 2022; Richard et al., 2016; Stumpf et al., 2017; Wiesner et al., 2018) and reprogramming (Guillemin et al., 2019; Ye et al., 2020). In addition, entropy analysis was recently used to describe the heterogeneity of chromatin states at developmentally regulated loci, as an effective information content-predicting local genome topology and the competence for binding transcription factors (D'Oliveira Albanus et al., 2021). Here, consistent with stochastic gene expression (Xu et al., 2021), we identified a high entropy of chromatin features among plant iPSC ancestors. This is reminiscent of mouse and human iPSC cultures displaying a high level of transcriptome and epigenetic heterogeneity, which is thought to correlate with functional heterogeneity (Cahan and Daley, 2013; Yokobayashi et al., 2021).

Interestingly, entropy decreases over culturing time (5–40% depending on features) suggesting a process orienting the trajectory of chromatin changes towards homogeneisation, although entropy remains largely positive after 5–7 days culturing. We found that entropy reduction is attenuated in the absence of phytohormones. Although it cannot be excluded that this is a result of higher cell viability in the presence of hormones, it is conceivable that phytohormone-based signalling directly influences chromatin modifiers and remodellers, with an effect on cell identity maintenance and cellular plasticity (Maury et al., 2019), and is promoted when cells are exposed to an inhibitor of histone deacetylation. In contrast, nutrient availability did not influence the heterogeneity of chromatin features. Interestingly, during erythroid cell differentiation, stochasticity in gene expression is increased in the presence of a drug inhibiting histone acetylation (Guillemin et al., 2019), rather than deacetylation, as in our case. Possibly, the heterogeneity of chromatin patterns and that of transcription profiles are uncoupled to some extent, but more studies on the control of gene expression stochasticity in both animal and plant iPSC ancestor cultures remain necessary.

### Is chromatin entropy as functional determinant of pluripotency acquisition?

A link between chromatin dynamics and chromatin pattern heterogeneity with the actual ability of plant iPSC ancestors to reprogramme remains to be established. Indeed, only a small fraction of cells from a plant iPSC ancestor culture will further develop in a pluripotent cell mass with regenerative ability (Pasternak et al., 2020; Sugimoto et al., 2011). Interestingly, in an *Arabidopsis*, in a leaf-derived protoplast culture consisting of 85% mesophyll cells, and hence that is initially relatively homogenous, only 0.5% cells seem to contribute to an effective regeneration process (Xu et al., 2021). This is reminiscent from the situation in animal iPSC cultures, which show variable levels of cellular reprogramming (0.5–10%) depending on the tissue source and inductive method (Kalra et al., 2021; Romanazzo et al., 2020).

Pluripotency in animal iPSCs has been proposed to be an emerging property of an intrinsically entropic cellular system, rather than from a unique property at the single-cell level (MacArthur and Lemischka, 2013). In this conceptual framework, where pluripotency is a statistical property of a microstate system, uncommitted PSCs have weak regulatory constraints leading to a high entropy and stochasticity in gene expression and chromatin states (MacArthur and Lemischka, 2013). As differentiation progresses, more regulatory constraints apply and the heterogeneity of the cellular system diminishes (MacArthur and Lemischka, 2013). Our findings suggest a similar conceptual framework to explain the properties of plant iPSC ancestors (Fig. 8) – the release of cell-wall-free cells away from the tissue context might abolish regulatory constraints stabilising cellular identity and leading to a high heterogeneity in gene expression and chromatin organisation. Appropriate culturing conditions might progressively restore regulatory signals in the culture, reducing entropy and thereby stabilizing gene expression and chromatin organization patterns (Fig. 8). Interestingly in this process, we found that phytohormones, at least at the specific ratio and concentration used in our conditions, are dampening this process and nutrient availability does not affect entropy reduction. This suggests that intrinsic properties of plant iPSC ancestor cultures re-establish regulatory constraints reducing the entropy of the cellular system.

In addition, the multiscale heterogeneity of chromatin patterns (as captured by textures) is reminiscent of the finding that variations in local chromatin density underscore the differentiation competence of human embryonic stem cells (hESCs) (Golkaram et al., 2017). This heterogeneity, which is influenced by genomic contacts but also by DNA-free space in the nucleus – a variable intrinsically captured by textures in our cytological analysis – is proposed to reflect variable states of molecular crowding, which in turn controls transcriptional bursts and noise underlying cellular reprogramming (Golkaram et al., 2016, 2017).

### Conclusion

Our work opens new perspectives to understand *in vitro* cellular reprogramming and pluripotency in plants. Notably, it is interesting to consider a conceptual framework where cellular variability and the associated chromatin and transcription entropy act as possible driving forces during reprogramming (Fig. 8), and where dedifferentiation and pluripotency acquisition result from the property of a cellular system rather than that of single cells, as has been proposed for animal iPSC reprogramming (MacArthur and Lemischka, 2013). Furthermore, whether the regulated abundance and type of linker histones variants in plant cells also drive chromatin reorganisation and epigenome reprogramming during dedifferentiation and pluripotency acquisition *in vitro*, like in animal cells (Fig. 8), is an exciting question to investigate.

## MATERIALS AND METHODS

### Plant material and growth conditions

The *Arabidopsis thaliana* plant lines expressing fluorescently tagged H1 variants under their native promoters were as described previously (Rutowicz et al., 2019, 2015). To generate the dual chromatin reporter line expressing H1.2–GFP and H2.B–RFP the line *promH1.2::H1.2-GFP* (Rutowicz et al., 2015) was crossed with *promUBQ10::H2B-RFP* (von Wangenheim et al., 2016).

Seeds were surface sterilised and rinsed in sterile water before transferring onto the sterile germination medium [0.5× MS medium (Carolina Biological Supplies, USA), 1% agar (Bacto Agar, BD -Beckton Dickinson- company, USA)]. Seeds were placed on the medium at an ∼1 cm distance using toothpicks, stratified at 2 days at 4°C and grown for 3 weeks with a long day photoperiod (16 h, 22°C day, and 8 h, 18°C night) and light flux of ∼100 μM s^−1^ m^−2^.

### Protoplast preparation and culture

Protoplasts were isolated from *Arabidopsis* leaves based on published protocols (Li et al., 2014; Yoo et al., 2007) with some modifications to ensure sterile conditions during isolation and protoplast culturing as described thereafter. The whole procedure was performed under a laminar hood. All solutions were filter sterilised using 0.22-μm filters. Blades, forceps white pieces of paper and tubes were autoclaved. For pipetting, sterile filter tips were used. After leaf tissue digestion, protoplasts were filtered with sterile single-use cell strainers with 70 μm pores. After isolation cells were suspended in the intended medium (Table S3) and distributed into, 96-well plates with coverglass bottoms (Greiner Bio-One, Ref: 655087) with 100 μl culture per well. The outer wells of the plate were excluded due to the limited field of view and travel range at imaging. Each plate was sealed with 3M tape to avoid drying, wrapped in a layer of aluminium foil and placed in the growth chamber for 5 to 7 days depending on the experiment.

For the trichostatin A (TSA) treatment, 100 nM or 200 nM TSA in DMSO (Sigma-Aldrich) was added at day 0 in the culture or the equivalent amount of DMSO (2% or 4%, Mock). To assess cell viability, fluorescein diacetate (FDA; Sigma-Aldrich) was added at either day 0, day 2 or day 5 before imaging.

### Microscopy imaging

Microscopy images shown in Fig. 1 were taken with a laser scanning confocal microscope (Leica SP5, Leica microsystems, Germany). For scoring (Fig. 1 graphs) the percentage of cells expressing the chromatin markers were scored manually by a researcher who was not aware of the experimental conditions under an epi-fluorescence microscope (Leica DM6000 Leica microsystems, Germany).

For all other figures, leaf protoplasts cultured in coverglass-bottom 96-well plates were imaged using a confocal microscope Cell Voyager (CV7000, Yokogawa), equipped with a 60× water immersion objective (Nikon, NA1.2) using illumination by 100–200 mW lasers from Coherent (depending on the channel) and filters from Chroma. Images were acquired as 16-bit images using two Neo sCMOS cameras (Andor).

For each well, six regions of interest (ROIs) corresponding to a field of view of 277 µm×234 µm with an image format of 2560×2160 pixels over 16 *z*-planes with a step of 1 µm were assessed. ROIs were randomly chosen without overlap. The imaging time for one well was 90 s; the imaging time for one plate (60 wells) was 90 min.

### Image analysis

Images were loaded into TissueMAPS (http://tissuemaps.org; code available at https://github.com/pelkmanslab/TissueMAPS) where ROIs were grouped as 2×3 grids per well. Illumination correction was based on averaged intensity statistics across all images and maximum intensity projection along the *z*-dimension was performed in TissueMAPS.

Image processing was undertaken in TissueMAPS v. 0.6.3 for the following steps: (i) gaussian smoothing with a filter-size of 5 pixels; (ii) Otsu thresholding in a user-defined range using the red (H2B–RFP) channel, followed by binary mask filling and filtering objects <200 pixels in area to get segmentation; (iii) feature measurements using the measure_morphology, measure_intensity and measure_texture modules in TissueMAPS for both channels without the smoothing applied (see Table S2); (iv) classification of mis-segmentations by interactive training of Support Vector Machine (SVMs) in TissueMAPS. For the training, two classes of objects were annotated and created: A, correctly segmented and B, non-correctly segmented. Then for each class ∼40–50 objects were labelled manually. Object morphology and RFP and GFP intensity features were chosen for training the classifier. In particular, the following parameters: area, circularity, roundness, elongation, convexity, mean intensity in RFP channel and mean intensity in GFP channel were used. In total, 100 objects in 10 images (total=1000) were used for training and resulted in 95% segmentation accuracy (*n*=417), with 4% of the 5% false positives being truncated nuclei at the edge of the image and 1% corresponding to undersegmented nuclei (Fig. S8). The description of the SVM algorithm which was used here is available at https://scikit-learn.org/stable/modules/svm.html). Features measurements and object classification were then downloaded from TissueMAPS for further analysis. In the R software environment (https://www.r-project.org/), we further filtered away outlier nuclei with an area >5000 pixels [‘giant nuclei’, 1–5% per dataset, possibly from trichomes (Walker et al., 2000)].

### Principal component analysis

The PCA was undertaken using Clustvis (Metsalu and Vilo, 2015). The original dataset exported from TissueMAPS (HTI001, HTI002, HTI004 or HTI005; see Table S4) was subset to remove non informative columns such as redundant identifier codes (related to the plate, experiment and objects), position information (such as Morphology_local centroid_X and _Y, well_position, is_border) and features or experiment description not relevant for the analysis. If too big for upload, the subset data was entered using the input type ‘paste data’; the data matrix was transposed (‘data matrix reshape/transpose matrix’) and the option ‘detect column and row annotations’ was unchecked to be adjusted manually. Columns with similar annotations were collapsed by taking the median inside each group. Unit variance scaling was applied to rows. SVD with imputation was used to calculate the principal components. The *x*- and *y*-axis show the principal component 1 and principal component 2, respectively, which explain the given percentage of the total variance. Prediction ellipses depict a 95% confidence interval (a new observation from the same group will fall inside the ellipse with probability *P=*0.95).

### Entropy analysis

The initial script for computing Shannon Entropy is described in Dussiau et al. (2022) and is available at https://osf.io/9mcwg/. The adapted script for computing entropy of chromatin features is provided at https://github.com/barouxlab/ChromatinEntropy. When all cells (segmented nuclei) express the same value for a given feature, this entropy of the feature will be null. The more cell-to-cell variability for a given chromatin feature, the higher value of entropy.

### Plots and statistical tests

Box plots, violin plots, scatter plots, 2D contours and histograms were created using online tools (https://chart-studio.plotly.com/, http://shiny.chemgrid.org/boxplotr/; Spitzer et al., 2014) or own R scripts (Fig. S1 only; script available upon request). Statistical tests indicated in the figure legends were done using the R package or using the online tool https://statskingdom.com/mean-tests.html.

## Supplementary Material



10.1242/joces.261703_sup1 Supplementary information

Table S1.
