# Supplementary material for: Multiscale chromatin dynamics and high entropy in plant iPSC ancestors
Source: J Cell Sci. 2024 Jun 24;137(20):jcs261703. doi: 10.1242/jcs.261703 (PMC11234377; doi:10.1242/jcs.261703)
Supplement: Supplementary information [file joces-137-261703-s1.pdf]

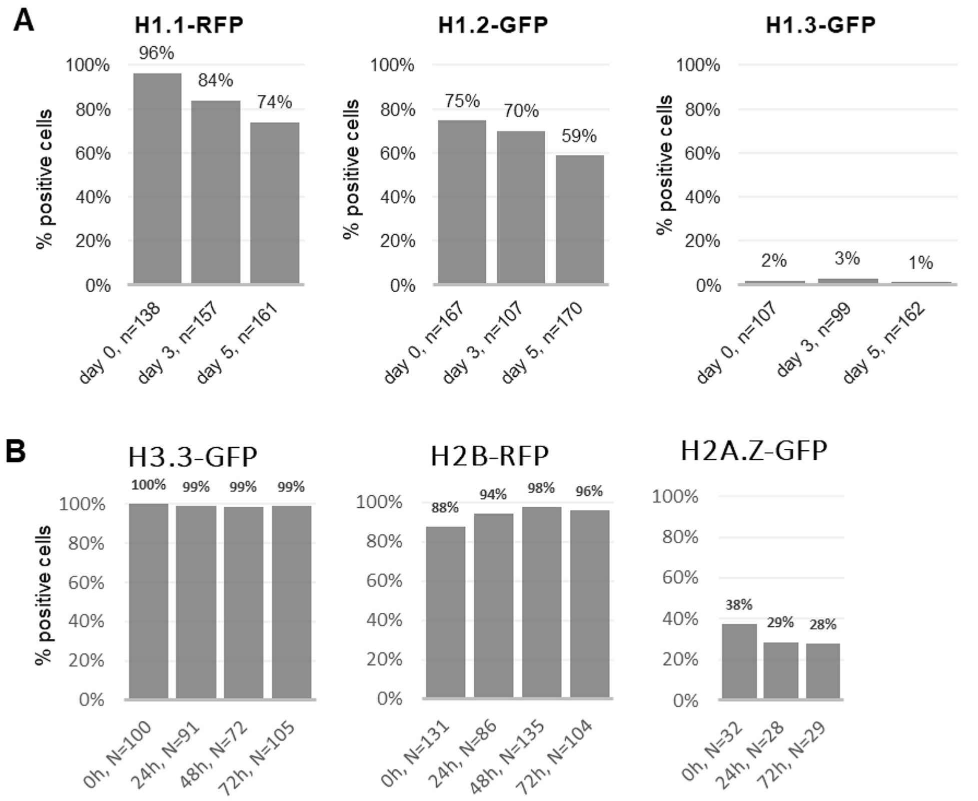

C % cells expressing the S-G2 cell cycle marker H3.1-mCherry

	# positive cells	total (n)	%
**day 0**	15	275	**5%**
**day 1**	15	271	**6%**
**day 2**	31	326	**10%**
**day 5**	20	250	**8%**
**day 6**	26	230	**11%**

Fig. S1. Expressivity of different histone variants and a cell cycle marker in leaf protoplast cultures

(A-B) Percentage of leaf protoplast showing detectable signal of histone markers (fluorescently tagged histone variants as indicated, see main text and methods for reference and details), at culturing days indicated along the X axis. N= number of cells scored. **(C)** Percentage of cells expressing an S-G2 cell cycle marker H3.1-mCherry (Desvoyes et al., 2020) during protoplast culturing. See also data source in Table S1.

Journal of Cell Science • Supplementary information

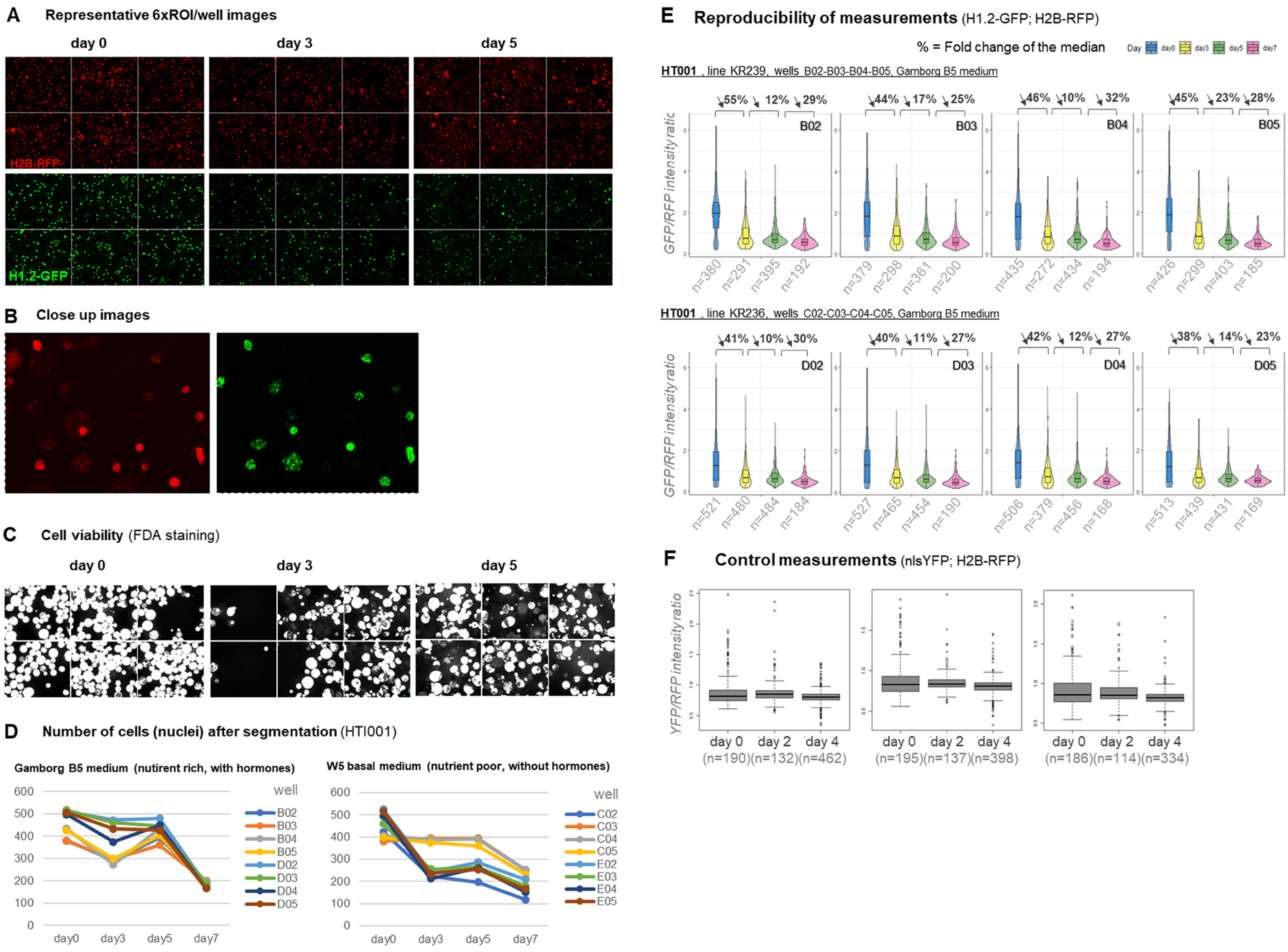
A Representative 6xROI/well images
day 0
day 3
day 5
H2B-RFP
H1.2-GFP
B Close up images
C Cell viability (FDA staining)
day 0
day 3
day 5
D Number of cells (nuclei) after segmentation (HTI001)
Gamborg B5 medium (nutrient rich, with hormones)
W5 basal medium (nutrient poor, without hormones)
well
B02 B03 B04 B05 D02 D03 D04 D05
C02 C03 C04 C05 E02 E03 E04 E05
day0 day3 day5 day7
E Reproducibility of measurements (H1.2-GFP; H2B-RFP)
% = Fold change of the median
HT001 , line KR239, wells B02-B03-B04-B05, Gamborg B5 medium
HT001 , line KR236, wells C02-C03-C04-C05, Gamborg B5 medium
GFP/RFP intensity ratio
F Control measurements (nlsYFP; H2B-RFP)
YFP/RFP intensity ratio
day 0 day 2 day 4
(n=190)(n=132)(n=462)
(n=195)(n=137)(n=398)
(n=186)(n=114)(n=334)

Fig. S2. Representative images from leaf protoplasts expressing the dual H1.2-GFP; H2B-RFP markers and cell viability data

(A) Overview of 6 regions of interests (ROIs) per well at day 0, 3 and 5. **(B)** zoom-in images of protoplasts. **(C)** Cell viability assessment at day 0, 3 and 5 with FDA staining. **(D)** Number of cells detected after segmentation showing a reduction towards day 7 of culturing, probably indicating a loss of viability. Comparison of cultures in two different media – the nutrient-rich Gamborg B5 medium and the nutrient-poor W5 basal medium (see Table S3). **(E)** Intensity ratio in protoplasts measured in four replicative wells at day 0, 3, 5 and 7 for two independent transgenic lines expressing H1.2-GFP;H2B-RFP. Arrow: decrease in % in the intensity ratio between two time points. (F) Intensity ratio at day 0, 2 and 4 in protoplasts co-expressing the control, nuclear-localised free YFP (nlsYFP) and H2B-RFP. Measurements for three replicates are shown. n, number of protoplasts analysed.

Journal of Cell Science • Supplementary information

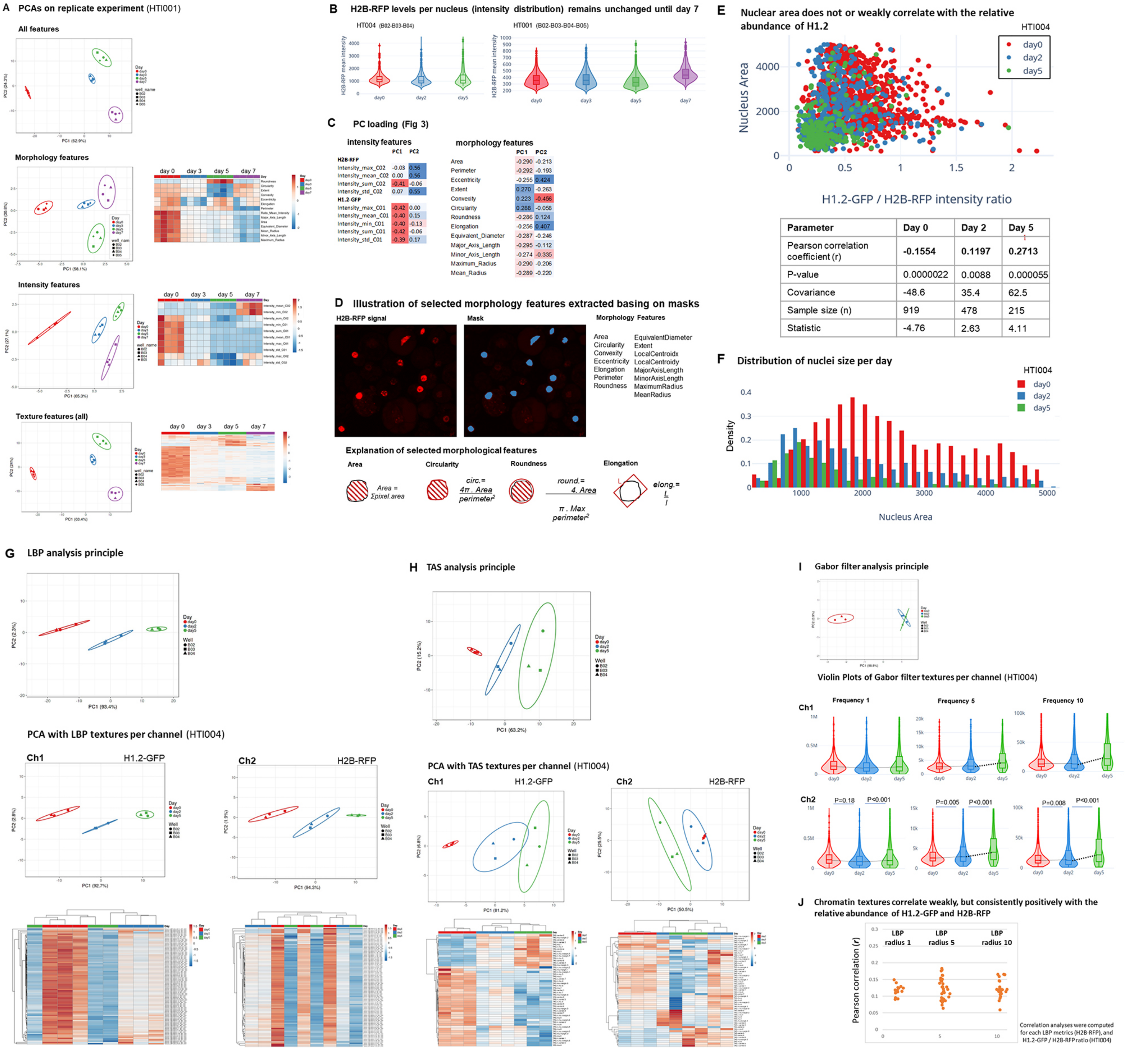
A PCAs on replicate experiment (HTI001)
All features
Morphology features
Intensity features
Texture features (all)
B H2B-RFP levels per nucleus (intensity distribution) remains unchanged until day 7
C PC loading (Fig 3)
intensity features
morphology features
D Illustration of selected morphology features extracted basing on masks
H2B-RFP signal
Mask
Morphology Features
Explanation of selected morphological features
Area
Circularity
Roundness
Elongation
E Nuclear area does not or weakly correlate with the relative abundance of H1.2
Nucleus Area
H1.2-GFP / H2B-RFP intensity ratio
Parameter | Day 0 | Day 2 | Day 5
Pearson correlation coefficient (r) | -0.1554 | 0.1197 | 0.2713
P-value | 0.0000022 | 0.0088 | 0.000055
Covariance | -48.6 | 35.4 | 62.5
Sample size (n) | 919 | 478 | 215
Statistic | -4.76 | 2.63 | 4.11
F Distribution of nuclei size per day
Density
Nucleus Area
G LBP analysis principle
PCA with LBP textures per channel (HTI004)
Ch1 H1.2-GFP
Ch2 H2B-RFP
H TAS analysis principle
PCA with TAS textures per channel (HTI004)
Ch1 H1.2-GFP
Ch2 H2B-RFP
I Gabor filter analysis principle
Violin Plots of Gabor filter textures per channel (HTI004)
Frequency 1
Frequency 5
Frequency 10
J Chromatin textures correlate weakly, but consistently positively with the relative abundance of H1.2-GFP and H2B-RFP
Pearson correlation (r)
LBP radius 1
LBP radius 5
LBP radius 10
Correlation analyses were computed for each LBP metrics (H2B-RFP), and H1.2-GFP / H2B-RFP ratio (HTI004)

Fig. S3. Analysis of morphology, intensity and texture features characterizing the protoplast chromatin, in replicate cultures and independent experiments

(A) Principal Component Analysis (PCA) computed on all features, on morphology features only, on intensity features only, or on LBP (Local Binary Pattern) texture features for H2B-RFP only, in protoplasts expressing H1.2-GFP and H2B-RFP at day 0, 3, 5 and 7; dataset HTI001. **(B)** Mean intensity signal for H2B-RFP in protoplasts during culturing in two different experiments HTI001 (sampling at day 0, 3, 5 and 7) and HTI004 (sampling at days 0, 2 and 5). **(C)** List of features contributing to the principal component presented in Figure 3; Blue indicates negative corelations and red indicates positive corelations. **(D)** Selected morphology features extracted from masks, left image – an example image illustrating H2B-RFP marked nuclei, right image – mask obtained based on the left image, bottom panel – illustration of the feature calculations. **(E)** Scatter plot and correlation analysis between nucleus area and H1.2-GFP;H2B-RFP intensity ratio in protoplast. **(F)** Density distribution of nucleus area in protoplast during culturing at day 0, day 2 and day 5. **(G)** PCA computed on the features belonging to the texture class LBP (Local Binary Pattern), bottom panels: PCA separately for each channel. **(H)** PCA computed on the features belonging to the texture class TAS (Threshold Applied Statistics), middle panels: PCA calculated separately for each channel; bottom panel: Heat map representing relative changes for TAS texture descriptors for the H1.2-GFP (left) and H2B-RFP (right) signal distribution. Heatmaps represent the median value for each descriptor, with unit variance scaling applied to rows. **(I)** PCA computed on the features belonging to the texture class Gabor (Gabor wavelet filter), middle and bottom panels showing plots of selective morphology descriptors (Frequencies 1, 5 and 10) for each channel separately; p-values calculated with Kruskal Wallis test followed by post-hoc Dunn's test and Bonferroni correction. **(J)** Pearson correlation analyses computed for selected LBP metrics (H2B-RFP), and H1.2-GFP / H2B-RFP ratio (data set: HTI004).

Additional information for PCA analyses: X and Y axis show principal component 1 and principal component 2 that explain the given % of the total variance, respectively. Ellipses: 95% confidence interval. Each point represent a culture replicate (well). Heatmaps represent the median value for each descriptor, with unit variance scaling applied to rows; Ch1 – channel detecting H1.2-GFP; Ch2 – channel detecting H2B-RFP; See list of features in Table S2.

Journal of Cell Science • Supplementary information

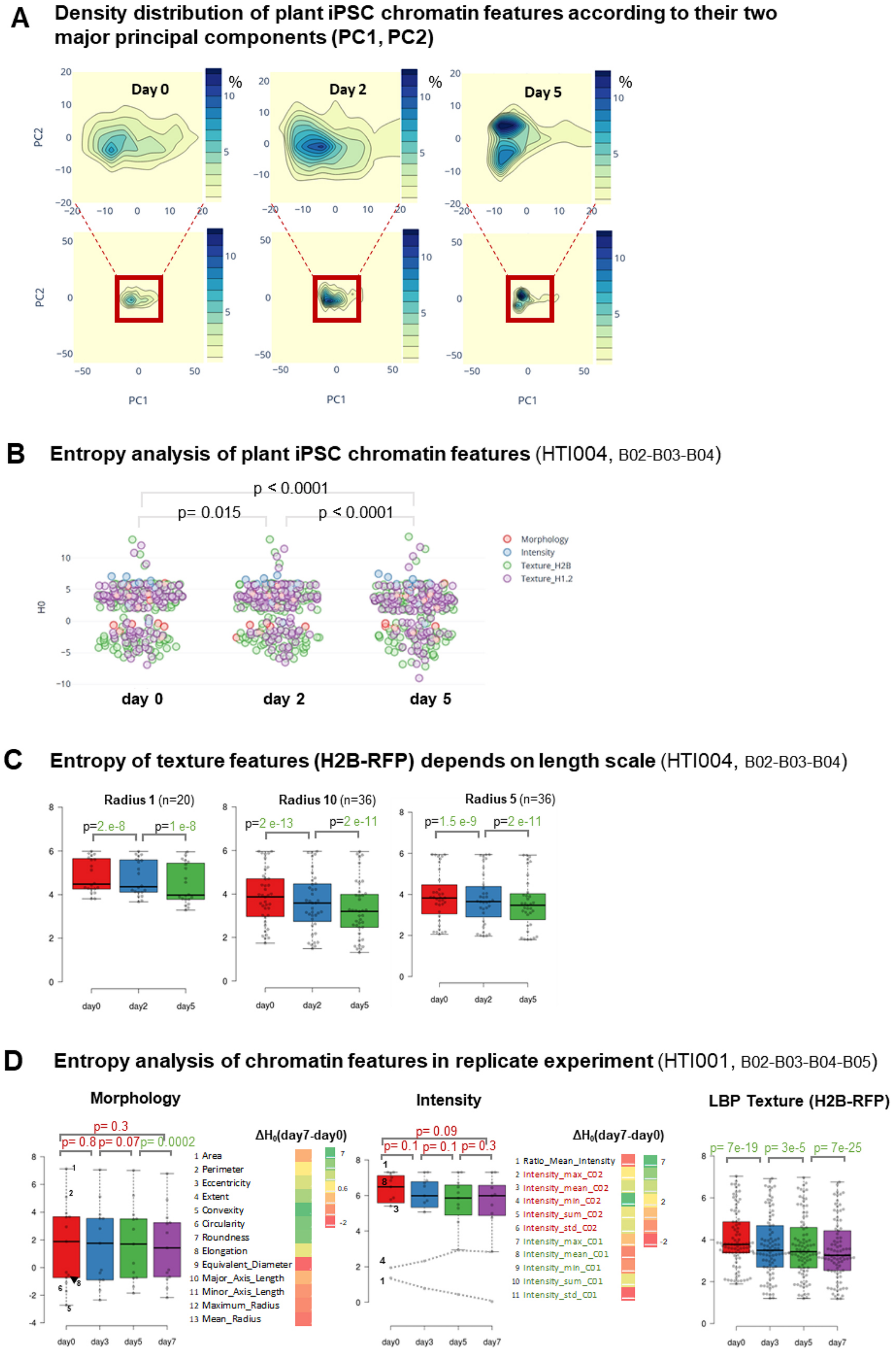

Fig. S4. (A) Density distribution of plant protoplast chromatin features according to their two major principal components (PC1, PC2). Density contours are coloured according to the frequency (percent) of nuclei falling in the corresponding PC space. **(B)** Entropy analysis of plant protoplast chromatin features during culturing; dataset HTI004; wells B02-B03-B04. **(C)** Entropy (H0) of texture features (H2B-RFP) for different length scales: Radius 1, 10 and 5, respectively; n, number of descriptors per family; dataset: HTI004; wells: B02-B03-B04. **(D)** Entropy (H0) of chromatin features per family; n, number of descriptors per family; Tukey whiskers extend to data points that are less than 1.5 x IQR away from 1st/3rd quartile. Analysed (those for which the distribution did not allow partitioning for entropy calculation were excluded; ΔH0, differential entropy; dataset HTI001. P-values calculated from paired t-test; n= number of features; see Table S2 for the list of features and main text for more information.

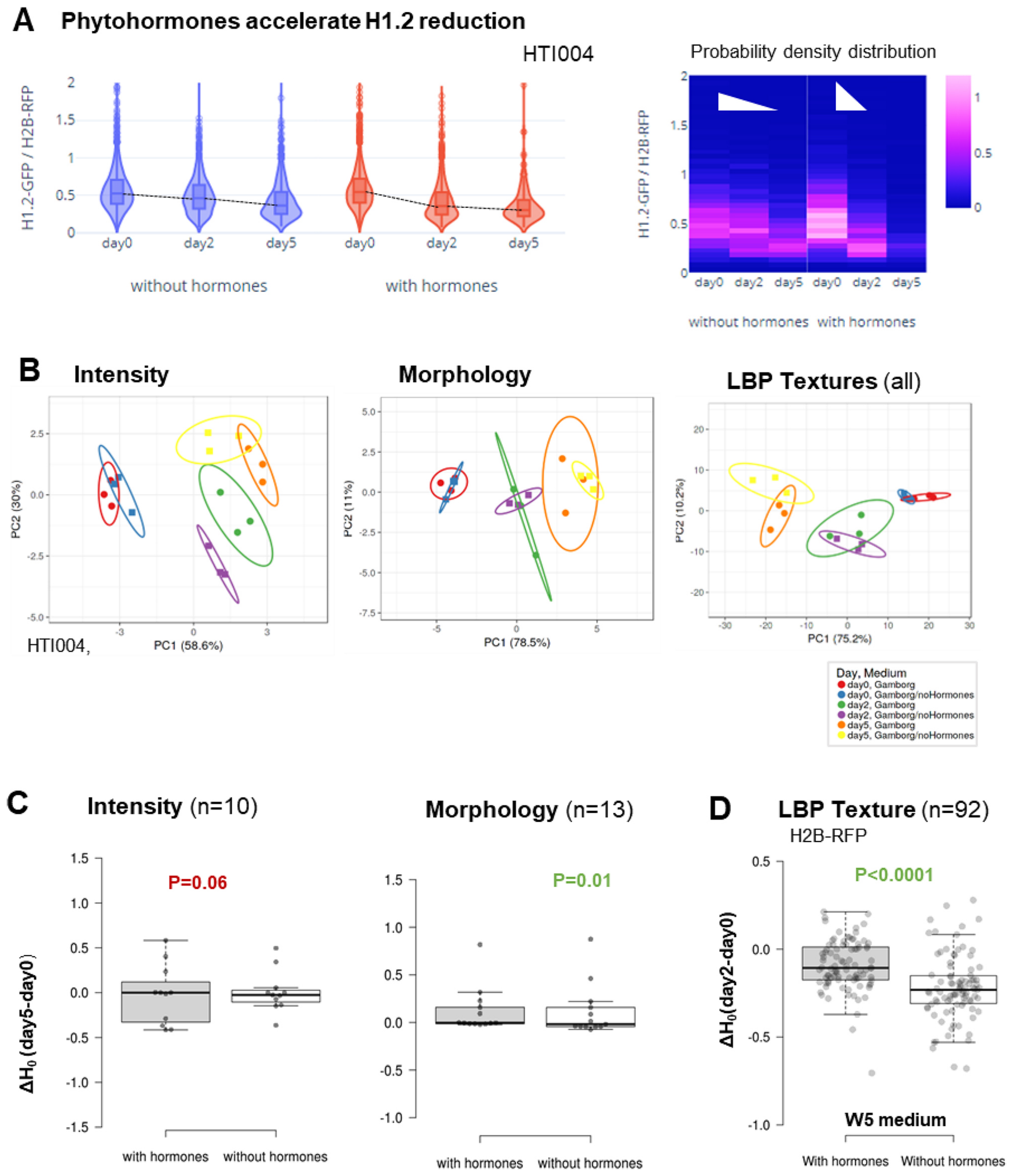

Fig. S5. Effect of phytohormones on chromatin features and their entropy in protoplast replicate cultures

(A) H1.2-GFP/H2B-RFP ratio in leaf protoplast cultures in the presence of absence of phytohormones showing a slightly accelerated decreased with phytohormones: dashed lines following the median on the left graph and, heatmap intensity distribution on the right panel **(B)** Principal Component Analysis (PCA) computed on intensity features, morphology features, and LBP texture features for H2B-RFP, respectively during protoplast culturing in the presence or absence of phytohormones. Colours indicate different days and treatment as indicated in the legend. **(C, D)** Differential entropy (ΔH0) between day 7 and day 0 for intensity and morphology features (C) and LBP textures (D), in leaf protoplast cultured in Gamborg (C) or W5 (D) in the presence or absence of phytohormones. n, number of features per feature group indicated. P value, paired t-test.

Journal of Cell Science • Supplementary information

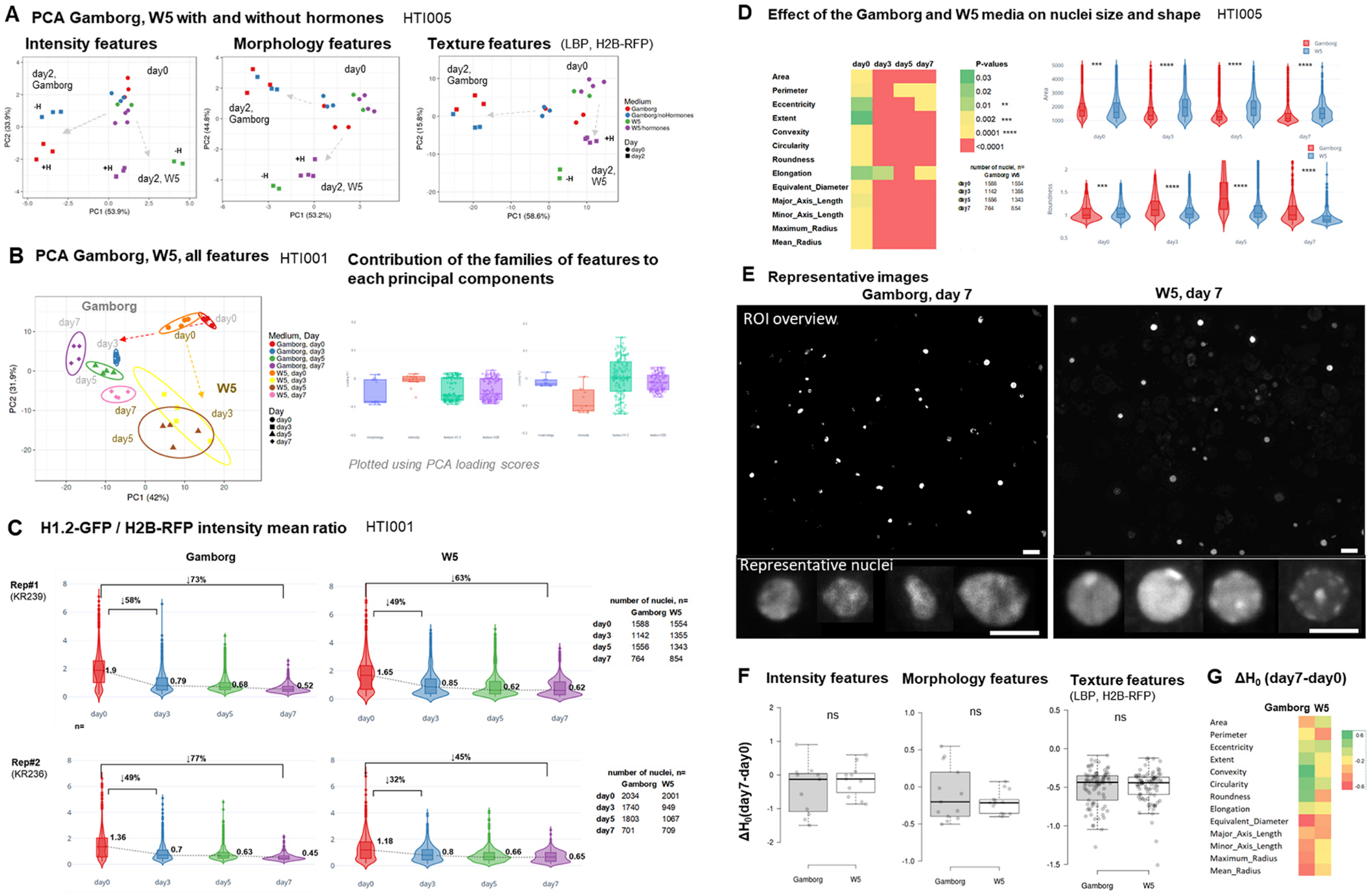

Journal of Cell Science • Supplementary information

Fig. S6. Effect of nutrients on chromatin features and their entropy in protoplast replicate cultures **(A)** Principal Component Analysis (PCA) computed on (i) intensity features, (ii) morphology features, (iii) texture features for H2B-RFP during protoplast culturing in nutrient rich (Gamborg) or nutrient poor (W5), each with hormones (+H) or without (-H). The cultures stem from the same, original pool of protoplast partitioned in the different media and imaged at day 0 and day 2 in two or three replicate wells (number of datapoints with the same colour); dataset HTI005. **(B)** PCA computed on all features during protoplast culturing in Gamborg and W5. The cultures stem from the same, original pool of protoplast partitioned in the different media and imaged at day 0, day 3, day 5 and day 7 in at least three replicate wells (number of datapoint with the same colour); right panel: contribution of the families of features to each principal components; dataset HTI001; Right panel: Contribution of the families of features to each principal component. **(C)** H1.2-GFP/H2B-RFP intensity mean ratio measured for protoplasts derived from two different Arabidopsis lines expressing H1.2-GFP/H2B-RFP. **(D)** Effect of the Gamborg and W5 media on nuclei size and shape; P-values Kruskal-Wallis test – all morphology features; left panel: heatmap showing the P value between day 7 and day 0 for each morphology features and pointing out the features with more drastic changes. Right panel: Violin plots for area and roundness. **(E)** Representative images of protoplast nuclei in two different media, sampled at day 7 of culturing, whole ROI and representative nuclei below. **(F)** Differential entropy $\Delta H0$ between day 7 and day0 for the set of intensity features, morphology features or LBP texture features, respectively, comparing protoplast cultured in the nutrient-rich Gamborg medium or nutrient-poor W5 basal medium. Ns, no significant difference (paired t-test). **(G)** Heatmap showing the P-value (paired t-test) comparing the differential entropy for each single morphology feature and showing a significant effect of nutrients on selective features only (red, orange values)

Journal of Cell Science • Supplementary information

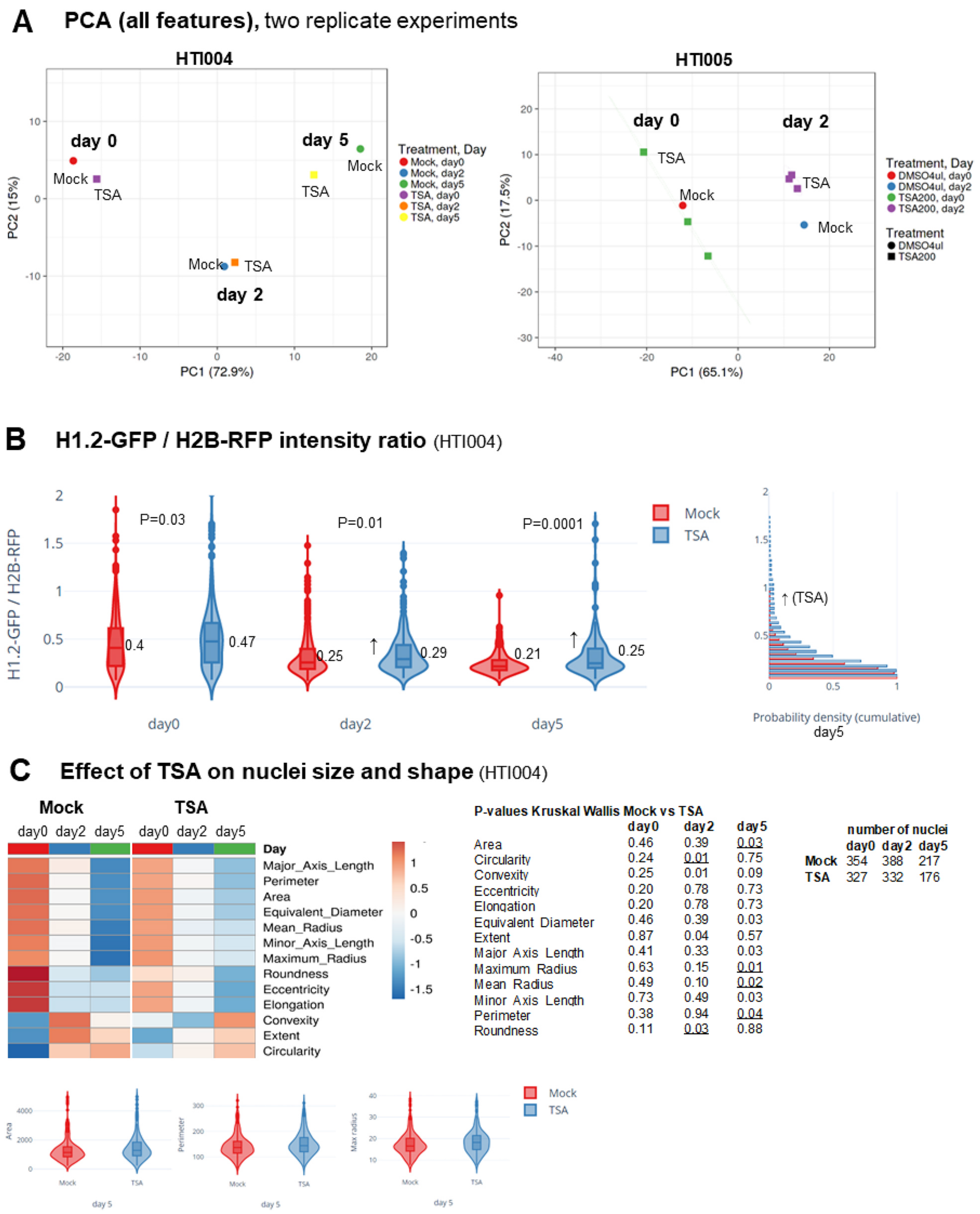

Fig. S7. Effect of the histone deacetylase inhibitor TSA on chromatin features and their entropy in protoplast cultures

(A) Principal Component Analysis (PCA) computed on all features for two datasets HTI004 and HTI005; PC1, PC2 - two major principal components; TSA, Trichostatin A (100 nM); Mock – 2% DMSO which was the solvent for TSA; TSA200 – double concentration of TSA (200 nM); Mock – 4% DMSO – double concentration of DMSO. **(B)** Left panel: violin plots for H1.2-GFP:H2B-RFP intensity ratio during protoplast culturing for TSA treatment; right panel: density plot for H1.2-GFP:H2B-RFP intensity ratio at day 5 for TSA treatment. **(C)** Effect of TSA on nuclei size and shape. Left panel: heatmap representing the median value for selected shape descriptors for protoplasts during culturing, treated with TSA and Mock; right panel: p-values calculated with Kruskal Wallis test for selected shape descriptors.

Journal of Cell Science • Supplementary information

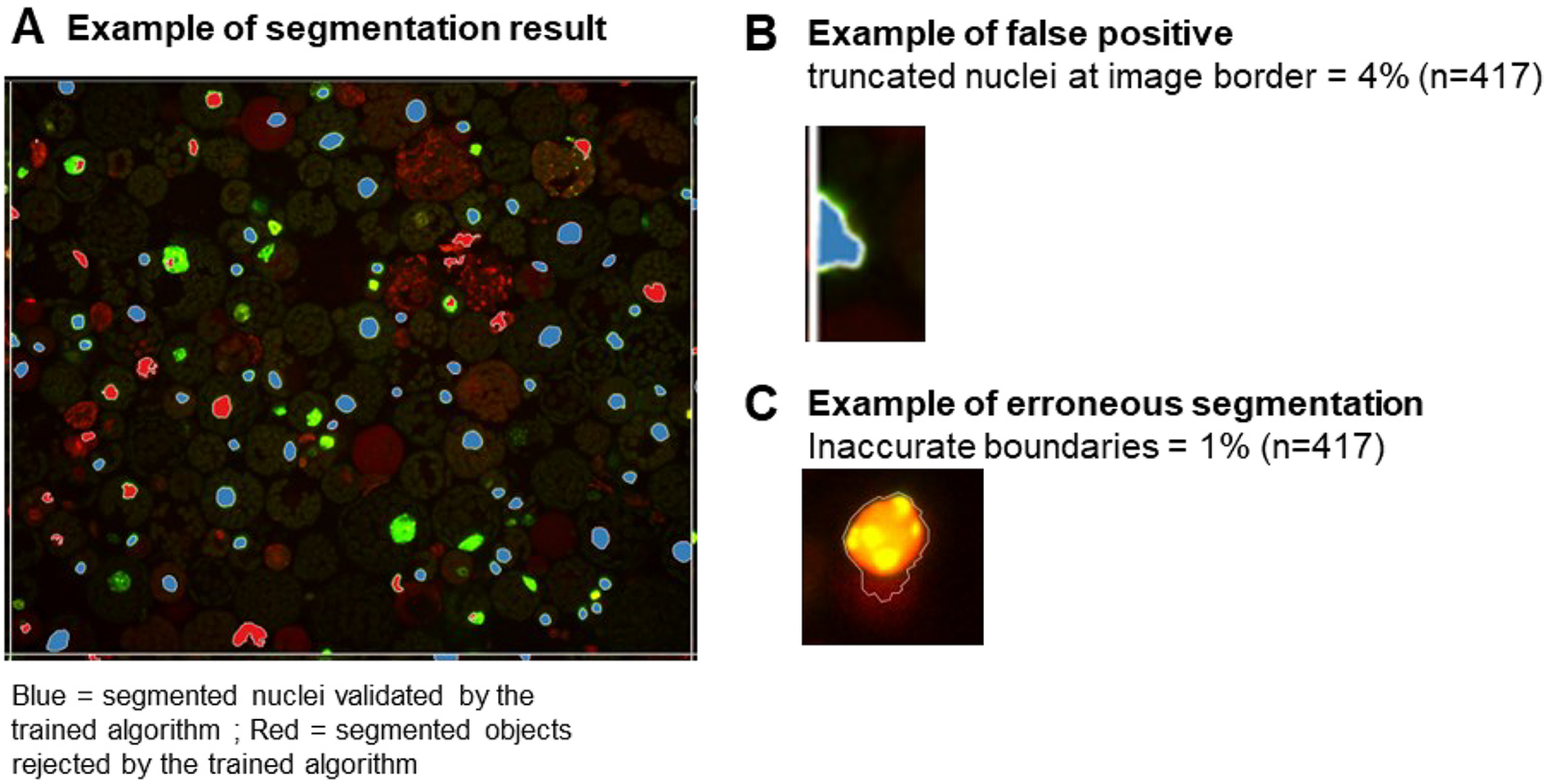

Fig. S8. Representative example of nuclei segmentation following training, based on the H2B-RFP signal

(A) Example of segmentation result; blue = segmented nuclei validated by the trained algorithm; red = segmented objects rejected by the trained algorithm. **(B)** Example of the false positive result of the object classification. **(C)** Example of erroneous segmentation.

References

Desvoyes, B., Arana-Echarri, A., Barea, M. D., & Gutierrez, C. (2020). A comprehensive fluorescent sensor for spatiotemporal cell cycle analysis in Arabidopsis. *Nat Plants*, **6**(11), 1330-1334.

Journal of Cell Science • Supplementary information

Table S1.

Available for download at
https://journals.biologists.com/jcs/article-lookup/doi/10.1242/jcs.261703#supplementary-data

Table S2. Summary of image features exported

**Morphology (15)**	**Intensity (5 per channel + 2)**	**Texture (4 methods, 172 metrics per channel)**			
Area	Intensity_max	**_LBP method (108)**	**_TAS (54)**	**_Hu (7)**	**_Gabor method (3)**
Circularity	Intensity_mean	radius 1-10.9 in steps of 0.01 to 0.1	center, mu, mu.margi, n.center, n.mu, n.mumargin radius from 0 to 8	steps 0 to 6	frequency 1, 5, 10
Convexity	Intensity_min				
Eccentricity	Intensity_sum				
Elongation	Intensity_std				
EquivalentDiameter	Channel Ratio_Mean_Intensity				
Extent	Channel Ratio_Sum_Intensity				
LocalCentroidx					
LocalCentroidy					
MajorAxisLength					
MinorAxisLength					
MaximumRadius					
MeanRadius					
Perimeter					
Roundness					

Table S3. Media composition.
Gamborg B5 Basal - Contains the macro- and micronutrients, and vitamins as described by Gamborg, et al. (1968).

	**Basic media**		Modified media			
	**W5**	**Gamborg**	W5 + hormones	Gamborg without hormones	Gamborg2	Gamborg2 without hormones
**Reagents**	[ml]	[ml] or [g]	[ml]	[ml] or [g]	[ml] or [g]	[ml] or [g]
Total volume	*50*	*50*	*50*	*50*	*50*	*50*
5M NaCl	1.54		1.54			
2M KCl	0.125		0.125			
1M $CaCl_2$	6.25	0.34	6.25	0.34	0.147	0.147
0.2M MES	0.5	0.8	0.5	0.8	0.8	0.8
Ampicilin	0.05	0.05	0.05	0.05	0.05	0.05
2,4D (1mg/ml)		0.05	0.05	0.05	0.05	
BAP (1.5mg/ml)		0.005	0.005	0.005		
Kinetin (1 mg/ml)					0.005	
Gamborg B5 Basal		0.16 g		0.16 g	0.16 g	0.16 g
Glucose		3.6 g		3.6 g	2 g	2 g
Mannitol					3 g	3 g

Journal of Cell Science • Supplementary information

Table S4. Datasets used for the study

Experiment	Imaging days	Replicate cultures (wells)	Culturing medium	Treatment	Line number	Genotype (transgene)
HTI001	day0, day3, day5, day7	B02-B05	Gamborg	none	KR239	*promH1.2::H1.2-GFP ; promUBQ10::H2B-RFP*
		C02-C05	W5	none	KR239	*promH1.2::H1.2-GFP ; promUBQ10::H2B-RFP*
		D02-D05	Gamborg	none	KR236	*promH1.2::H1.2-GFP ; promUBQ10::H2B-RFP*
		E02-E05	W5	none	KR236	*promH1.2::H1.2-GFP ; promUBQ10::H2B-RFP*
HTI002	day0, day2, day4	B06-B07	Gamborg	none	KR318	*promHTR5::YFPnls ; promUB10::H2B-RFP*
		B09-B10	Gamborg	none	KR317	*promHTR5::YFPnls ; promUB10::H2B-RFP*
		C05-C07	W5	none	KR317	*promHTR5::YFPnls ; promUB10::H2B-RFP*
		C08-C10	W5	none	KR318	*promHTR5::YFPnls ; promUB10::H2B-RFP*
HTI004	day0, day2, day5	B2-B4	Gamborg	none	KR239	*promH1.2::H1.2-GFP ; promUBQ10::H2B-RFP*
		B5	Gamborg	FDA at day0	KR239	*promH1.2::H1.2-GFP ; promUBQ10::H2B-RFP*
		B6	Gamborg	FDA at day2	KR239	*promH1.2::H1.2-GFP ; promUBQ10::H2B-RFP*
		B7	Gamborg	FDA at day5	KR239	*promH1.2::H1.2-GFP ; promUBQ10::H2B-RFP*
		B8-B10	Gamborg	TSA 100nm	KR239	*promH1.2::H1.2-GFP ; promUBQ10::H2B-RFP*
		B11	Gamborg	DMSO 2%	KR239	*promH1.2::H1.2-GFP ; promUBQ10::H2B-RFP*
		C2-C4	Gamborg/noHormones	none	KR239	*promH1.2::H1.2-GFP ; promUBQ10::H2B-RFP*
HTI005	day0, day2	D2-D4	W5/with_hormones	none	KR239	*promH1.2::H1.2-GFP ; promUBQ10::H2B-RFP*
		D8	Gamborg	TSA 100 nM at day1	KR239	*promH1.2::H1.2-GFP ; promUBQ10::H2B-RFP*
		D9-D10	Gamborg	none	KR239	*promH1.2::H1.2-GFP ; promUBQ10::H2B-RFP*
		D11	Gamborg	DMSO 2% at day1	KR239	*promH1.2::H1.2-GFP ; promUBQ10::H2B-RFP*
		E4	W5	none	KR239	*promH1.2::H1.2-GFP ; promUBQ10::H2B-RFP*
		E5	W5	none	KR239	*promH1.2::H1.2-GFP ; promUBQ10::H2B-RFP*
		E9-E10	Gamborg	TSA 200nM	KR239	*promH1.2::H1.2-GFP ; promUBQ10::H2B-RFP*
		E11	Gamborg	DMSO 4%	KR239	*promH1.2::H1.2-GFP ; promUBQ10::H2B-RFP*
