# Supplementary material for: Multiscale chromatin dynamics and high entropy in plant iPSC ancestors
Source: J Cell Sci. 2024 Jun 24;137(20):jcs261703. doi: 10.1242/jcs.261703 (PMC11234377; doi:10.1242/jcs.261703)
Supplement: Supplementary file 1 [file joces-137-261703_review_history.pdf]

Multiscale chromatin dynamics and high entropy in plant iPSC ancestors

Kinga Rutowicz, Joel Lüthi, Reinoud de Groot, René Holtackers, Yauhen Yakimovich, Diana M. Pazmiño, Olivier Gandrillon, Lucas Pelkmans and Célia Baroux
DOI: 10.1242/jcs.261703

Editor: Guillaume Jacquemet

Review timeline

Original submission:	10 October 2023
Editorial decision:	22 December 2023
First revision received:	20 March 2024
Editorial decision:	5 April 2024
Second revision received:	24 April 2024
Accepted:	29 April 2024

Original submission

First decision letter

MS ID#: JOCES/2023/261703

MS TITLE: Multiscale chromatin dynamics and high entropy in plant iPSC cultures

AUTHORS: Kinga Rutowicz, Joel Luethi, Reinoud de Groot, Rene Holtackers, Yauhen Yakimovich, Diana M. Pazmino, Olivier Gandrillon, Lucas Pelkmans, and Celia Baroux
ARTICLE TYPE: Research Article

We have now reached a decision on the above manuscript.

To see the reviewers' reports and a copy of this decision letter, please go to: https://submit-jcs.biologists.org and click on the 'Manuscripts with Decisions' queue in the Author Area. (The corresponding author only has access to reviews.)

As you will see, one of the reviewers, raised several substantial criticisms that prevent me from accepting the paper at this stage. They suggest, however, that a revised version might prove acceptable, if you can address their concerns. If you think you can deal satisfactorily with the criticisms on revision, I would be pleased to see a revised manuscript. We would then return it to the reviewers.

Please ensure that you clearly highlight all changes made in the revised manuscript. Please avoid using 'Tracked changes' in Word files as these are lost in PDF conversion.

I should be grateful if you would also provide a point-by-point response detailing how you have dealt with the points raised by the reviewers in the 'Response to Reviewers' box. Please attend to all of the reviewers' comments. If you do not agree with any of their criticisms or suggestions, please explain clearly why this is so.

Reviewer 1

Advance summary and potential significance to field

© 2024. Published by The Company of Biologists under the terms of the Creative Commons Attribution License (https://creativecommons.org/licenses/by/4.0/).

In this manuscript, Kinga Rutowicz and colleagues established a semi-automated pipeline for high-throughput quantitative analysis of chromatin reporters at the single cell level, allowing a detailed analysis of the level of heterogeneity during plant protoplast culture. This work provides new perspectives to understand cell fate transition.

Comments for the author

In this manuscript, Kinga Rutowicz and colleagues described dynamic chromatin patterns of protoplasts isolated from Arabidopsis leaves during 7 days-culturing through image analysis of multiple chromatin features. Overall, they summarized that nutrients availability played important roles in regulating chromatin organization and heterogeneity was controlled by the culturing environment and intrinsic epigenetic factors during iPSC culturing. However, the data provided by authors does not support the conclusions well.

Major points:

Protoplasts isolated from Arabidopsis leaves and cultured for 7 days were regarded as plant iPSC, which is not rigorous. Generally, it took 2 or 3 months to reprogram Arabidopsis somatic cells (eg, mesophyll protoplast) to reacquire pluripotency. The authors are not supposed to draw conclusions about plant iPSC without proving the pluripotency of the plant protoplasts.
The correlation between H1.2 abundance and chromatin changes of iPSC should not be established only based on the data that H1.2-GFP showed 30% decrease of GFP abundance and H2B was equally expressed throughout culturing. The expression of H1.2 and H2B in callus derived from Arabidopsis leaves are needed.

In addition, development potential of protoplast showing different H1.2 abundance at day 7 should be evaluated by combining Flow Cytometry and regeneration experiments.

In Figure 5, the entropy seemed to decrease more significantly in the absence of phytohormones. The authors need to rule out the influence of phytohormones on cell viability and iPSC efficiency.

Giving the moderate effect of TSA treatment on H1.2 : H2B ratio and nuclei shape, the data about effect of TSA treatment on Arabidopsis iPSC culture and the histone acetylation dynamic during iPSC induction is needed to provided.

Minor points:
H1.2 reduction still took place without phytohormones did not suggest H1.2 reduction may not be initiated by culturing (line 293-295).
At least three biological repeats and t-test are required to compare W5 with Gamborg B5.

<u>Reviewer 2</u>

Advance summary and potential significance to field

The study is of high quality and the analytical strategy is well described. The full set of data and the availability of the scripts are very much appreciated.
The discussion extends the interest of this study to animal iPSC which is of great interest.

Comments for the author

The manuscript entitled “Multiscale chromatin dynamics and high entropy in plant iPSC cultures” by Rutowicz et al., reports a high throughput image analysis protocol to decipher chromatin dynamics in protoplasts.

© 2024. Published by The Company of Biologists under the terms of the Creative Commons Attribution License (https://creativecommons.org/licenses/by/4.0/).

The authors used this tool to measure several nuclear and chromatin during a time course following protoplasts preparation. For this, reporter constructs of particular histones variants have been tested and chosen to serve as marker to measure chromatin dynamics.

In addition, the authors identified that chromatin changes depend predominantly on nutrient availability rather than phytohormones.

This study provides important analytical tools and reports very nice results for a general audience.

First revision

Author response to reviewers' comments

We thank the Reviewers for their careful reading and for their evaluation of our manuscript.

We are pleased to read that Reviewer 2 is very positive about the study and does not seem to have concerns. Reviewer 1 also acknowledges that “This work provides new perspectives to understand cell fate transition “which let us to understand that our study is of interest for the community. However, Reviewer 1 expressed some concerns that we addressed carefully.

Notably, we realized that a major concern arises from our semantic choice of “plant iPSC” which created some confusion. We propose a text revision that should address and clarify this issue. We also added references throughout the text and a discussion paragraph to better place our work in context of the knowledge particularly in animal iPSC systems.

We provide our point-by-point response below detailing the revisions, which we hope will satisfy the reviewers and editor.

In addition, please note that while revising, some changes were made in the text as follows:

- Linguistic improvements (minor changes in a sentence)

- Discussion: the first text part was modified to clarify the scope of the study in context of plant cell reprogramming and also in relation to reviewer 1 comment on plant iPSC definition

- New discussion paragraph entitled “A role of linker histone in plant cell dedifferentiation?”: it is not an entirely new text. We transferred a part originally in the section “A rapid, multiscale reorganisation of chromatin which trajectory depends on nutrient availability and less on phytohormones” relative to H1 dynamics, and we added some discussion in light of the knowledge in the animal field

- New paragraph entitled “conclusive remarks” summarizing the main findings and proposing an illustrated working model in the new Figure 8

- New Figure 8 also enabling to clarify the position of the study in relation to reviewer 1 comment

We provide a version with the changes highlighted in yellow. We also reformatted the supplemental figures as requested by the editorial office.

Reviewer 1 Advance Summary and Potential Significance to Field:
In this manuscript, Kinga Rutowicz and colleagues established a semi-automated pipeline for high-throughput quantitative analysis of chromatin reporters at the single cell level, allowing a detailed analysis of the level of heterogeneity during plant protoplast culture. This work provides new perspectives to understand cell fate transition.

Major points:

© 2024. Published by The Company of Biologists under the terms of the Creative Commons Attribution License (https://creativecommons.org/licenses/by/4.0/).

1) Protoplasts isolated from Arabidopsis leaves and cultured for 7 days were regarded as plant iPSC, which is not rigorous. Generally, it took 2 or 3 months to reprogram Arabidopsis somatic cells (eg, mesophyll protoplast) to reacquire pluripotency. The authors are not supposed to draw conclusions about plant iPSC without proving the pluripotency of the plant protoplasts.

Authors' response:
We agree that our semantic choice was confusing.

Plant pluripotent stem cells are largely associated in the literature with the expression of *WUS* and *WOX5* in the meristem, among others. Consequently, it is considered that *in vitro* only the cells expressing these typical markers correspond to plant (shoot-type) iPSC (Reviewed by Sang et al "iPSCs: A Comparison between Animals and Plants", doi: 10.1016/j.tplants.2018.05.008). In vitro, the expression of genes associated with meristem (pluripotent stem cell) fate appear between 11 and 22 days in Arabidopsis (micro)calli (for instance, Xu et al 2021 doi: 10.1126/sciadv.abg8466), which is the stage qualifying for pluripotency as defined above.

Our initial semantic choice, however, came from the proposal that protoplasts are "stem-like" cells (Pasternak et al 2020 doi: 10.3390/ijms21124195 and Grafi 2004 doi: 10.1016/j.ydbio.2003.12.027) and the idea that they can produce pluripotent cell masses.

But after careful consideration, we agree with the reviewer that protoplast are not the iPSC yet, rather, the ancestor pool of cells from which some pluripotent cells will be derived.

We thus thank the reviewer for raising this important point and we propose, in the new manuscript, to keep the term protoplast in most instances, but to use the term "plant iPSC ancestors" in some places notably when discussing commonalities/differences with animal iPSC ancestors (starting cultures), in order to foster conceptual comparisons. Corrections have been made in the abstract, main text and legends accordingly.

Also, in a new Figure 8, we propose a model based on our findings and previous concepts from the literature emphasing cellular heterogeneity. In this scheme, the proper plant iPSC are clearly depicted as specific cells appearing in callus masses later in the culturing process.

We thank the reviewer for enabling this important clarification.

2) The correlation between H1.2 abundance and chromatin changes of iPSC should not be established only based on the data that H1.2-GFP showed 30% decrease of GFP abundance and H2B was equally expressed throughout culturing. The expression of H1.2 and H2B in callus derived from Arabidopsis leaves are needed.

Authors' response:

Based on the semantic correction we proposed, we think the problem is resolved. Our study focuses on the reprogramming phase prior to iPSC fate establishment. Studying the chromatin state of individual cells in calli would be an entire new work and beyond the scope of this study.

3) In addition, development potential of protoplast showing different H1.2 abundance at day 7 should be evaluated by combining Flow Cytometry and regeneration experiments.

Authors' response:

We agree that this would be an interesting study and again, an entire new work. But since we now propose a semantic correction, and again, because this study is focused on the dedifferentiation and reprogramming phase of the culture, and not on the regeneration process itself, this experiment is beyond the scope of this study.

© 2024. Published by The Company of Biologists under the terms of the Creative Commons Attribution License (https://creativecommons.org/licenses/by/4.0/).

4) In Figure 5, the entropy seemed to decrease more significantly in the absence of phytohormones. The authors need to rule out the influence of phytohormones on cell viability and iPSC efficiency.

Authors’ response:

The entropy is calculated on viable cells only. Besides, as shown in Supplemental 2D, the number of segmented, viable cells decreases over time more quickly in the absence of phytohormones. Thus, whether entropy decrease among the surviving cells is the result of selection by cell death of a selective (more variable) population, or due to direct influence of phytohormones on chromatin reorganisation cannot be determined. We now clarified this in the text also using a new reference (Maury et al, 2029, Phytohormone and Chromatin Crosstalk: The Missing Link For Developmental Plasticity? doi: 10.3389/fpls.2019.00395).

In the results:

“This suggests that phytohormones contribute maintaining a certain level of heterogeneity during dedifferentiation, in the plant iPSC ancestor cultures. Whether the effect is direct, with chromatin reorganisation responding to phytohormones, or indirect, due to higher cell viability in the presence of phytohormones (Supplemental Figure 1D) remains to be determined. Yet, the first scenario is supported by the numerous evidence of crosstalk between phytohormones and chromatin modifiers particularly affecting plant cell identity and plasticity (Maury et al, 2019).

In the discussion

“Although it cannot be excluded that it is a result of higher cell viability in the presence of hormones, it is conceivable that phytohormone-based signalling directly influence chromatin modifiers and remodellers, with an effect on cell identity maintenance and cellular plasticity (Maury et al., 2019).”

5) Giving the moderate effect of TSA treatment on H1.2 : H2B ratio and nuclei shape, the data about effect of TSA treatment on Arabidopsis iPSC culture and the histone acetylation dynamic during iPSC induction is needed to provided.

Authors’ response:

We thank the author for this suggestion. In Choi et al. (2021, doi: 10.3390/ijms22157817), an effect of TSA on protoplasts was found already at 100nM to increase histone acetylation and promote chromatin decondensation. Here we used 200nM hence we are in the functional range. In addition, whether a quantification would show a strong, moderate or low reduction of acetylation will not modify the conclusion that histone acetylation influences chromatin re-patterning over time in culture, as measured by the entropy metrics. A western blot or cytological measurement may not be sensitive to detect bulk changes but this would not exclude an effect. Indeed, for instance in Choi et al 2023 (10.1371/journal.pone.0279627) 100nM TSA did not give a significant change in histone acetylation levels in western blot analyses but was very effective to increase the division rate in lettuce protoplasts. Thus, instead, we plan to test more epigenetic drugs with different concentration range, duration of treatment and combination in a future study. But here, we aimed at benchmarking the whole system and provide insights into potential discoveries, based on concentrations already shown to affect the levels of histone acetylation. We hope that this perspective and explanation satisfies the reviewer.

6) Minor points:

H1.2 reduction still took place without phytohormones did not suggest H1.2 reduction may not be initiated by culturing (line 293-295).

© 2024. Published by The Company of Biologists under the terms of the Creative Commons Attribution License (https://creativecommons.org/licenses/by/4.0/).

Authors’ response: This is correct. Thank you for this comment. We now changed the sentence for “This suggests that H1.2 reduction is not a response to phytohormones in the medium but most likely a response to the cellular isolation, away from the source tissue, and culturing”

At least three biological repeats and t-test are required to compare W5 with Gamborg B5.

Authors’ response: If the reviewer refers to the data shown Figure 6A and supplemental Figure 6A, we have 3 replicates for all conditions except for W5, due to a problem in one well. But we have three replicates for W5 with hormones. Both W5 replicates and the three W5 with phytohormones replicates separate at day 2 from the Gamborg’s samples. Thus, altogether five W5 replicates against six Gamborg’s replicate, which we believe are sufficient to conclude. We would also like to point out a replicate experiment shown supplemental Figure 6B, with four replicates for each W5 and Gamborg’s conditions. Altogether, we believe the number of replicate experiments and replicate cultures in each are enough to conclude.

Regarding the statistical tests, Figure 6D shows a statistical evaluation for the nuclear size parameters. If other variables should be tested please instruct.

*****

Reviewer 2 Advance Summary and Potential Significance to Field

The study is of high quality and the analytical strategy is well described. The full set of data and the availability of the scripts are very much appreciated. The discussion extends the interest of this study to animal iPSC which is of great interest.

Reviewer 2 Comments for the Author

The manuscript entitled “Multiscale chromatin dynamics and high entropy in plant iPSC cultures” by Rutowicz et al., reports a high throughput image analysis protocol to decipher chromatin dynamics in protoplasts.

The authors used this tool to measure several nuclear and chromatin during a time course following protoplasts preparation. For this, reporter constructs of particular histones variants have been tested and chosen to serve as marker to measure chromatin dynamics. In addition, the authors identified that chromatin changes depend predominantly on nutrient availability rather than phytohormones. This study provides important analytical tools and reports very nice results for a general audience.

Second decision letter

MS ID#: JOCES/2023/261703

MS TITLE: Multiscale chromatin dynamics and high entropy in plant iPSC ancestors..

AUTHORS: Kinga Rutowicz, Joel Luethi, Reinoud de Groot, Rene Holtackers, Yauhen Yakimovich, Diana M. Pazmino, Olivier Gandrillon, Lucas Pelkmans, and Celia Baroux
ARTICLE TYPE: Research Article

We have now reached a decision on the above manuscript.

To see the reviewers' reports and a copy of this decision letter, please go to: https://submit-jcs.biologists.org and click on the 'Manuscripts with Decisions' queue in the Author Area. (Corresponding author only has access to reviews.)

© 2024. Published by The Company of Biologists under the terms of the Creative Commons Attribution License (https://creativecommons.org/licenses/by/4.0/).

As you will see, the reviewer raised some critical points that will require amendments to your manuscript. I hope that you will be able to carry these out because I would like to be able to accept your paper, depending on further comments from reviewers.

Please ensure that you clearly highlight all changes made in the revised manuscript. Please avoid using 'Tracked changes' in Word files as these are lost in PDF conversion.

I should be grateful if you would also provide a point-by-point response detailing how you have dealt with the points raised by the reviewers in the 'Response to Reviewers' box. Please attend to all of the reviewers' comments. If you do not agree with any of their criticisms or suggestions please explain clearly why this is so.

Reviewer 1

Advance summary and potential significance to field

In this manuscript, Kinga Rutowicz and colleagues established a semi-automated pipeline for high-throughput quantitative analysis of chromatin reporters at the single cell level, allowing a detailed analysis of the level of heterogeneity during plant protoplast culture. This work provides new perspectives to understand cell fate transition.

Comments for the author

Major points:
Since the authors proposed the protoplast cultures to be iPSC ancestor, the evidence proving that the protoplasts can acquire pluripotency during culturing should be provided.
minor points:
Callus/pluripotent cell masses can be induced from arabidopsis shoot or root explants directly by culturing in callus-inducing medium, meaning that it is not necessary to remove the cell wall before the calli induction.
The authors are not supposed to emphasize to get protoplasts to induce pluripotent cells in line 17, 18 58 and 59.

Reviewer 2

Advance summary and potential significance to field

Please see my first review

Comments for the author

No more comments

Second revision

Author response to reviewers' comments

Response to Reviewers

Reply to Reviewer 1

Major points:
Since the authors proposed the protoplast cultures to be iPSC ancestor, the evidence proving that the protoplasts can acquire pluripotency during culturing should be provided.

© 2024. Published by The Company of Biologists under the terms of the Creative Commons Attribution License (https://creativecommons.org/licenses/by/4.0/).

Reply from authors.
As explained in the manuscript and in the last response to the reviewers, the protoplasts have been long proposed to be stem-like cells with the possibility to reprogram towards pluripotent cells. While some consider protoplasts already dedifferentiated and pluripotent, others consider that pluripotency is only achieved at the callus stage, if not only for a few cells expressing meristem specific markers. While in the first manuscript version, we adhered to the permissive view, following Reviewer 1's comments we modified the text towards the last, most restrictive view, and wrote a new paragraph now lines 57-64 referring to state-of-the-art reviews in the field. We also clearly explain that this process is inefficient, as it in in animals. The work presented in this manuscript does not have the aim to reproduce all previous work demonstrating the properties of protoplasts, instead we focused on new aspects regarding chromatin organisation. It is worth noting that protoplasts were prepared and cultured here under very classical conditions following published protocols and media, as properly referenced in the manuscript. To further improve clarity, however, we modified the new sentences line 64 and thereafter for "Protoplast cultures, considered to share "stem cell-like" properties (Grafi et al., 2011; Sang et al., 2018) can thus be seen as the ancestors of plant iPSCs, similarly to the ancestor cultures of animal iPSCs, consisting in cells released from animal tissues and destinated for in vitro reprogramming. Also, these plant and animal ancestor cultures share the low efficiency (<0.5%) of iPSC production (Xu et al., 2021, Ghaedi & Niklason, 2019)."

minor points:
Callus/pluripotent cell masses can be induced from arabidopsis shoot or root explants directly by culturing in callus-inducing medium, meaning that it is not necessary to remove the cell wall before the calli induction.
The authors are not supposed to emphasize to get protoplasts to induce pluripotent cells in line 17, 18 58 and 59.

Reply of the authors
We agree with the reviewer that there are other ways to produce calli. The work is not focusing on this tissue, rather on the very early stages of dedifferentiation in culture.
We modified lines 17-18 "Plant protoplasts constitute the starting material to induce pluripotent cell masses" to ""Plant protoplasts provide a starting material to induce pluripotent cell masses" Lines 58-59 "Conveniently in plants, cells released from aerial, or underground tissues following an enzymatic degradation of the cell wall, protoplasts, provide starting material to generate pluripotent cells." was not at all excluding, as the reviewer seemed to think, other ways to derive pluripotent cell masses". Nevertheless, we modified the sentence for "Conveniently in plants, excised root or shoot fragments, as well as isolated cells devoid of cell wall (protoplasts) provide starting material to generate pluripotent cells (Ikeuchi et al, 2019)"

Third decision letter

MS ID#: JOCES/2023/261703

MS TITLE: Multiscale chromatin dynamics and high entropy in plant iPSC ancestors cultures.

AUTHORS: Kinga Rutowicz, Joel Luethi, Reinoud de Groot, Rene Holtackers, Yauhen Yakimovich, Diana M. Pazmino, Olivier Gandrillon, Lucas Pelkmans, and Celia Baroux
ARTICLE TYPE: Research Article

I am happy to tell you that your manuscript has been accepted for publication in Journal of Cell Science, pending standard ethics checks.

© 2024. Published by The Company of Biologists under the terms of the Creative Commons Attribution License (https://creativecommons.org/licenses/by/4.0/).
